# Supplementary material for: Clonal memory of colitis accumulates and promotes tumor growth
Source: bioRxiv. 2025 Feb 17:2025.02.13.638099. Preprint. [Version 1] doi: 10.1101/2025.02.13.638099 (PMC11870415; doi:10.1101/2025.02.13.638099)
Supplement: Supplement 1 [file media-1.pdf]

pattern	num_seqlets	modisco_cwm_fwd	modisco_cwm_rev	delta_effects	match0	qval0	match0_logo	match1	qval1	match1_logo	match2	qval2	match2_logo
de novo #1	37				Spi1	1.000000e+00		Spic	1.000000e+00		Erf	1.000000	
de novo #2	822				Dbx1	2.321360e-02		Pou4f1	2.321360e-02		Tlx1	2.321360e-02	
de novo #3	429				Zfp287	1.772060e-04		Foxj1	7.364640e-03		Onecut3	1.304850e-02	
de novo #4	1048				Zfp287	5.913510e-06		Prdm6	1.290500e-04		Zfp422	5.815600e-02	
de novo #5	598				Zfp287	9.573920e-08		Prdm6	1.070780e-06		Zfp422	1.100520e-04	
de novo #6	1448				Zfp422	2.528330e-04		Zfp287	6.258960e-02		Zfp182	1.212740e-01	
de novo #7	51				Onecut3	1.200830e-01		Zfp287	1.393100e-01		Onecut2	0.211331	
de novo #8	201				Stat5b	1.843740e-01		Stat5a	1.000000e+00		Nfatc2	1.000000e+00	
de novo #9	591				Foxj1	1.060450e-02		Zfp287	2.122260e-02		Ahctf1	2.127700e-02	
de novo #10	122				Stat5b	1.290560e-01		Nfatc2	1.290560e-01		Lcorl	0.129056	
de novo #11	502				Dbx1	9.037290e-03		Pou4f2	1.022360e-02		Tlx1	1.022360e-02	
de novo #12	3704				Zfp422	8.096640e-02		Zfp653	9.223390e-01		Hmga1	9.223390e-01	
de novo #13	2227				Zfp287	4.970410e-02		Prdm6	3.663160e-01		Zfp422	3.663160e-01	
de novo #14	189				Lcorl	9.101520e-02		Hmg20a	1.252270e-01		Hmg20b	1.252270e-01	
de novo #15	1002				Zfp422	4.450230e-01		Prdm6	4.450230e-01		Mef2c	5.741100e-01	
de novo #16	3152				Zfp287	2.526300e-10		Prdm6	7.235480e-06		Zfp422	9.543510e-06	

pattern	num_seqlets	modisco_cwm_fwd	modisco_cwm_rev	delta_effects	match0	qval0	match0_logo	match1	qval1	match1_logo	match2	qval2	match2_logo
de novo #17	4842				Zfp422	6.847590e-01		Hmga1	8.756110e-01		Hmga1rs1	8.756110e-01	
de novo #18	1782				Zfp422	6.325630e-03		Phf21a	1.527410e-01		Onecut3	1.558990e-01	
de novo #19	840				Prdm6	1.904400e-04		Zfp287	3.265320e-04		Zfp422	3.866320e-03	
de novo #20	53				Foxl1	1.673410e-02		Foxs1	1.673410e-02		Foxl2	2.029320e-02	
de novo #21	31				Foxl2	4.869560e-02		Foxl1	4.869560e-02		Foxs1	4.869560e-02	
de novo #22	84				Aire	7.374560e-01		Pdx1	7.374560e-01		Fosb	7.374560e-01	
de novo #23	654				Hmg20a	3.179440e-02		Hmg20b	3.179440e-02		Dbx1	3.179440e-02	
de novo #24	69				Nfatc2	9.180730e-01		Nfatc3	9.180730e-01		Hmga1	0.918073	
de novo #25	2928				Zfp287	6.254440e-02		Dmrtc2	3.953860e-01		Zfp422	3.953860e-01	
de novo #26	1811				Zfp422	4.094140e-03		Phf21a	2.559790e-02		Hmga1	8.141000e-02	
de novo #27	499				Zfp287	3.351640e-05		Foxj1	7.942150e-03		Onecut3	7.942150e-03	
de novo #28	1166				E2f3	7.450860e-02		E2f2	1.233520e-01		Hmga2	1.233520e-01	
de novo #29	1537				Zfp422	2.691930e-02		Hmga1	4.812690e-02		Hmga1rs1	4.812690e-02	
de novo #30	43				Lcorl	5.553020e-01		Lcor	6.489340e-01		Mef2a	1.000000	
de novo #31	1442				Zfp422	2.549470e-02		Phf21a	5.806890e-02		Onecut3	5.806890e-02	

pattern	num_seqlets	modisco_cwm_fwd	modisco_cwm_rev	delta_effects	match0	qval0	match0_logo	match1	qval1	match1_logo	match2	qval2	match2_logo
de novo #32	80				Foxb1	3.181580e-01		Foxb2	3.181580e-01		Foxa1	3.181580e-01	
de novo #33	7011				Zfp287	3.006580e-09		Prdm6	1.766260e-05		Zfp422	2.402780e-05	
de novo #34	181				Cdx2	1.408320e-05		Hoxb9	1.511210e-03		Hoxd10	0.001823	
de novo #35	219				Zfp287	1.243550e-05		Ahctf1	5.099930e-03		Onecut3	1.725870e-02	
de novo #36	2925				Zfp287	6.432160e-10		Prdm6	8.085120e-06		Zfp422	8.085120e-06	
de novo #37	70				Foxd3	1.228630e-02		Foxj3	1.228630e-02		Foxa2	1.228630e-02	
de novo #38	1832				Stat5b	1.000000e+00		Zbtb26	1.000000e+00		Onecut3	1.000000e+00	
de novo #39	178				Zfp287	1.047040e-08		Prdm6	2.810660e-06		Zfp422	0.000015	
de novo #40	84				Zfp287	1.087240e-03		Foxj1	1.419960e-02		Prdm6	1.798950e-01	
de novo #41	72				Arid3b	2.650430e-03		Hmga1	2.650430e-03		Hmga1rs1	0.002650	
de novo #42	995				Zfp422	8.619030e-05		Zfp287	3.300600e-03		Ahctf1	4.891120e-03	
de novo #43	5209				Zfp422	1.000000e+00		Arid3a	1.000000e+00		Stat5b	1.000000e+00	
de novo #44	5719				Zfp287	2.245740e-09		Prdm6	1.062610e-05		Zfp422	1.772310e-05	
de novo #45	103				Prdm6	7.685300e-04		Zfp287	1.149070e-03		Hmga1	1.415110e-01	
de novo #46	1932				Zfp287	2.865800e-09		Prdm6	1.761310e-06		Zfp422	1.124950e-04	

pattern	num_seqlets	modisco_cwm_fwd	modisco_cwm_rev	delta_effects	match0	qval0	match0_logo	match1	qval1	match1_logo	match2	qval2	match2_logo
de novo #47	540				Zfp287	1.013040e-05		Ahctf1	5.816930e-03		Onecut3	5.816930e-03	
de novo #48	56				Foxa1	5.033160e-04		Foxb1	5.033160e-04		Foxb2	0.000503	
de novo #49	1153				Stat5b	9.863860e-01		Hmga1	9.863860e-01		Hmga1rs1	9.863860e-01	
de novo #50	1043				Hmg20a	3.422490e-03		Hmg20b	3.422490e-03		Arid3b	4.663100e-02	
de novo #51	214				Zfp287	1.163460e-04		Prdm6	1.219970e-03		Zfp422	2.901820e-03	
de novo #52	845				Dbx1	1.599150e-02		Tlx1	2.179860e-02		Tlx2	2.179860e-02	
de novo #53	492				Zfp422	1.118690e-02		Zfp287	4.042720e-02		Hmga1	9.814870e-02	
de novo #54	149				Zfp287	8.284720e-04		Zfp879	2.646410e-02		Onecut3	0.031811	
de novo #55	4030				Zfp422	9.031700e-02		Hmga1	8.601250e-01		Hmga1rs1	8.601250e-01	
de novo #56	113				Hoxb9	3.496850e-02		Hoxd12	3.496850e-02		Cdx1	3.496850e-02	
de novo #57	543				Zfp287	1.432580e-06		Zfp422	2.500160e-05		Prdm6	4.701770e-05	
de novo #58	602				Zfp422	1.643460e-05		Zfp287	1.070010e-03		Prdm6	1.522820e-03	
de novo #59	138				Lcorl	2.937400e-02		Lcor	1.070470e-01		Stat5b	5.853570e-01	
de novo #60	1677				Zfp287	4.305550e-06		Zfp422	8.882290e-06		Prdm6	7.783480e-05	
de novo #61	102				Cdx2	6.084670e-04		Hoxd10	9.032200e-04		Hoxb9	2.316980e-03	

pattern	num_seqlets	modisco_cwm_fwd	modisco_cwm_rev	delta_effects	match0	qval0	match0_logo	match1	qval1	match1_logo	match2	qval2	match2_logo
de novo #62	2447				Zfp287	9.068750e-09		Prdm6	4.181820e-06		Zfp422	9.032410e-06	
de novo #63	1307				Zfp287	1.202760e-06		Prdm6	7.505940e-03		Zfp182	1.222340e-02	
de novo #64	733				Zfp422	1.755540e-02		Prdm6	1.755540e-02		Zfp287	1.755540e-02	
de novo #65	82				Foxj3	9.184690e-03		Foxc2	9.184690e-03		Foxa1	0.009185	
de novo #66	6447				Arid3a	9.083160e-01		Hmga1	9.083160e-01		Hmga1rs1	9.083160e-01	
de novo #67	859				Zfp422	2.730660e-05		Zfp287	2.117900e-04		Prdm6	1.114120e-02	
de novo #68	2727				Zfp287	3.946280e-01		Zfp422	3.946280e-01		Bcl6b	8.281590e-01	
de novo #69	3023				Hmga1	5.997750e-01		Hmga1rs1	5.997750e-01		Arid3b	5.997750e-01	
de novo #70	267				Hmg20a	1.896330e-02		Hmg20b	1.896330e-02		Phf21a	1.896330e-02	
de novo #71	121				Hmga1	9.657970e-04		Hmga1rs1	9.657970e-04		Tbp	2.017060e-03	
de novo #72	546				Zfp287	3.162490e-04		Prdm6	6.699180e-03		Zfp422	1.053430e-02	
de novo #73	112				Phf21a	4.855030e-01		Hmga1	4.855030e-01		Hmga1rs1	4.855030e-01	
de novo #74	456				Hmga1	5.958860e-01		Hmga1rs1	5.958860e-01		Phf21a	0.595886	
de novo #75	4534				Zfp287	1.291410e-09		Prdm6	6.742950e-06		Zfp422	1.313530e-05	
de novo #76	547				Zfp287	1.720780e-07		Prdm6	2.203830e-06		Zfp422	0.000016	

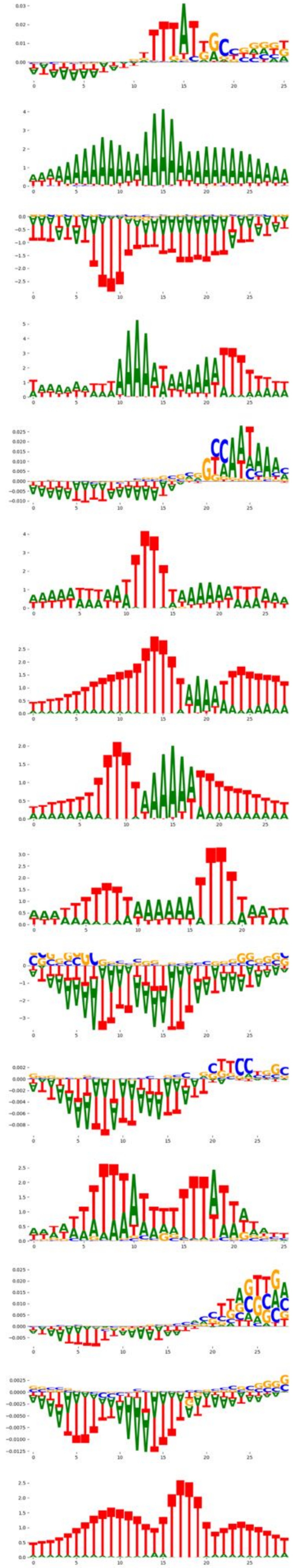
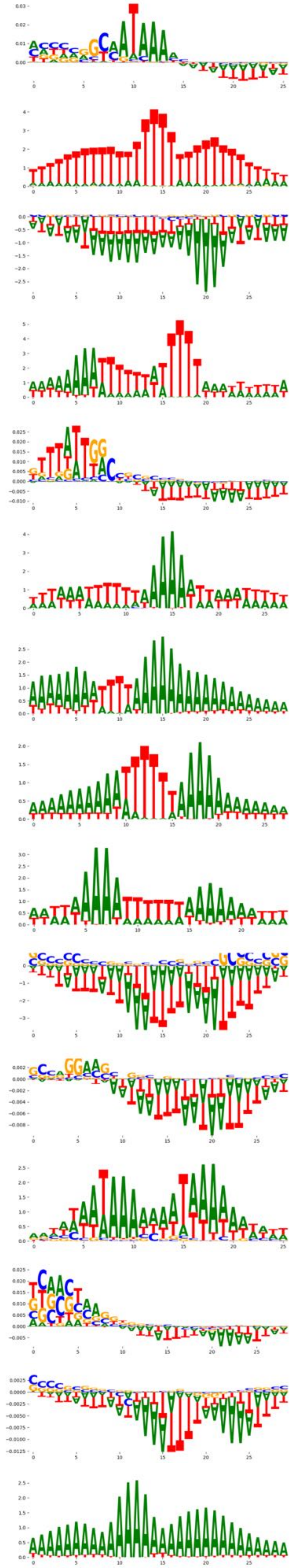
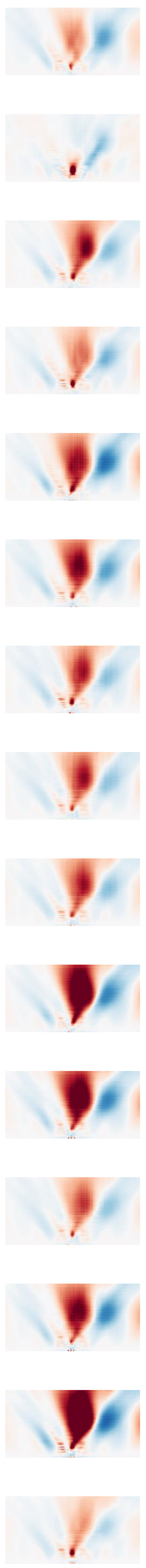
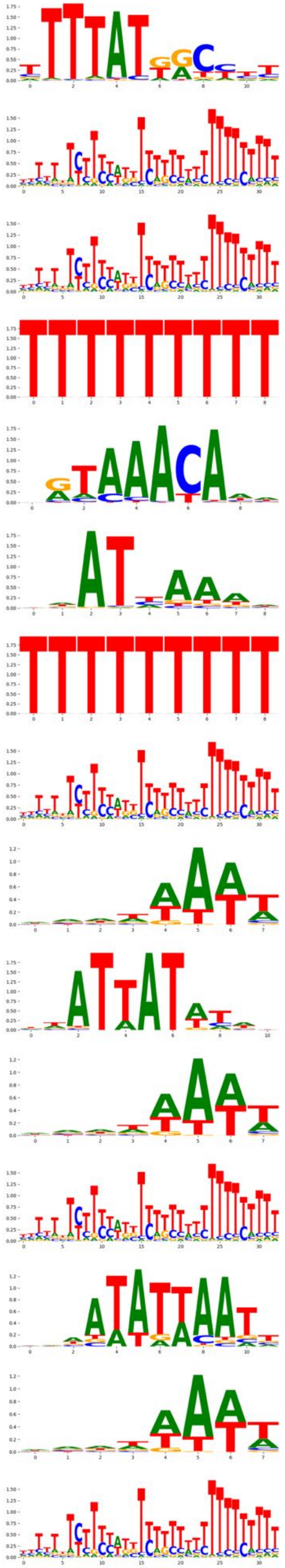
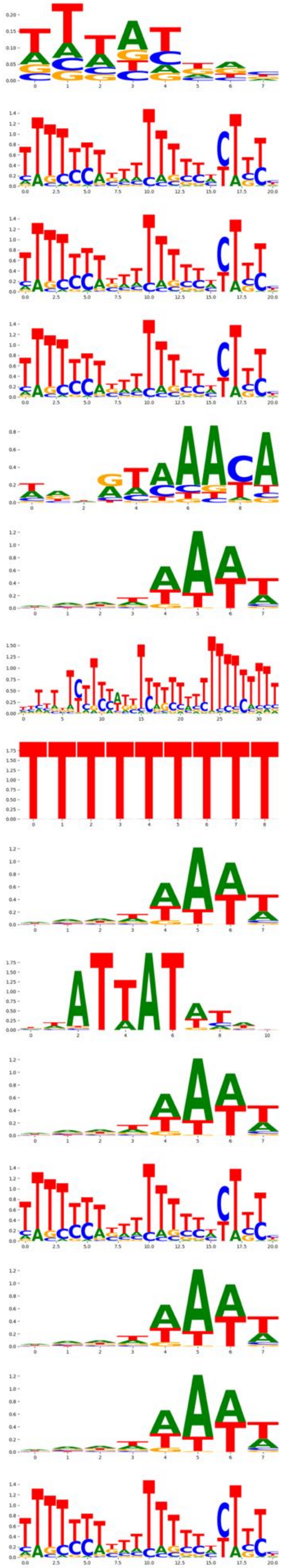
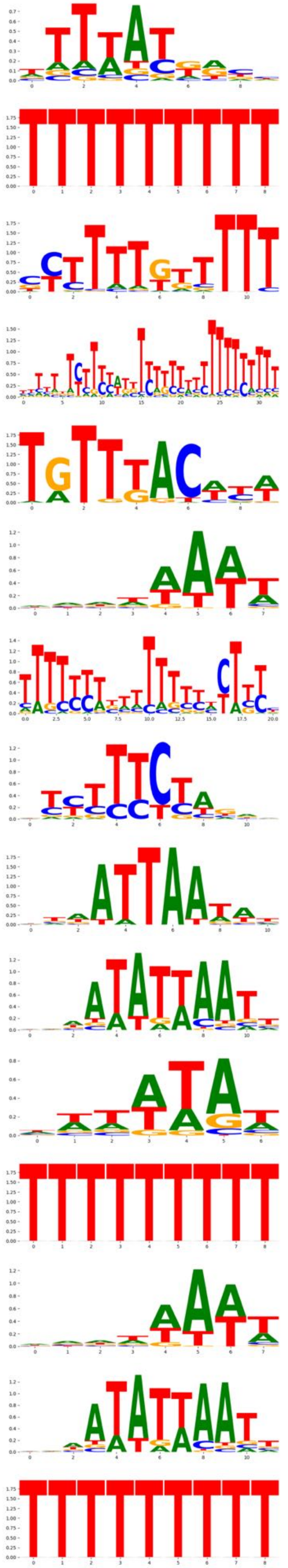

pattern	num_seqlets	modisco_cwm_fwd	modisco_cwm_rev	delta_effects	match0	qval0	match0_logo	match1	qval1	match1_logo	match2	qval2	match2_logo
de novo #77	74				Zfp879	2.667670e-01		Phf21a	2.667670e-01		Lcorl	0.266767	
de novo #78	33				Lcorl	1.635620e-01		Lcor	4.738640e-01		Mef2a	0.811895	
de novo #79	437				Zfp422	8.533530e-05		Zfp287	5.374730e-04		Ahctf1	1.078770e-01	
de novo #80	1869				Zfp287	5.241820e-02		Prdm6	7.345790e-02		Zfp422	2.182690e-01	
de novo #81	269				Prdm6	9.192990e-02		Kdm2b	9.192990e-02		Hmg20a	1.290550e-01	
de novo #82	14083				Fosl2	2.123640e-03		Fos	2.123640e-03		Bach2	2.794930e-03	
de novo #83	75				Fosb	2.758940e-01		Jund	2.758940e-01		Mga	2.758940e-01	
de novo #84	53				Fosb	1.000000e+00		Mafa	1.000000e+00		Mafk	1.000000e+00	
de novo #85	11965				Fosl2	3.390240e-04		Fos	3.381150e-03		Fosb	0.003381	
de novo #86	7678				Bach2	2.271530e-03		Fosl2	2.271530e-03		Fos	2.271530e-03	
de novo #87	28				Ppard	4.615580e-03		Nr2c2	4.615580e-03		Fosb	4.615580e-03	
de novo #88	210				Fosb	1.093940e-03		Fos	6.420790e-03		Jund	0.007821	
de novo #89	6318				Bach2	2.255230e-03		Fosl2	2.255230e-03		Fos	2.255230e-03	
de novo #90	9638				Fosl2	3.152090e-03		Fos	3.152090e-03		Bach2	3.614410e-03	
de novo #91	46				Mga	2.358490e-01		Tbx4	2.358490e-01		Tbx5	0.235849	

pattern	num_seqlets	modisco_cwm_fwd	modisco_cwm_rev	delta_effects	match0	qval0	match0_logo	match1	qval1	match1_logo	match2	qval2	match2_logo
de novo #92	7638				Bach2	2.659940e-03		Fosl2	2.659940e-03		Fosb	2.659940e-03	
de novo #93	56				Fosb	1.000000e+00		Mafa	1.000000e+00		Jund	1.000000e+00	
de novo #94	8313				Fosl2	3.216590e-03		Fos	3.216590e-03		Bach2	3.226330e-03	
de novo #95	63				Arntl	1.144040e-02		Tfe3	1.144040e-02		Tfeb	1.144040e-02	
de novo #96	10258				Fosl2	1.761910e-03		Fos	1.761910e-03		Bach2	2.495220e-03	
de novo #97	49				Fosb	9.999980e-01		Mafa	9.999980e-01		Tbx15	9.999980e-01	
de novo #98	108				Fosb	3.471140e-01		Jund	3.940460e-01		Junb	0.745869	
de novo #99	22				Fosb	1.290610e-04		Jund	6.236450e-02		Fosl2	7.204690e-02	
de novo #100	100				Hoxd11	8.195540e-02		Jund	8.195540e-02		Hoxc10	8.195540e-02	
de novo #101	26				Nr4a2	7.795930e-03		Nr4a3	7.795930e-03		Junb	1.529980e-01	
de novo #102	7800				Bach2	2.478920e-03		Fosl2	2.478920e-03		Fos	2.478920e-03	
de novo #103	71				Hoxd10	4.665250e-02		Hoxd11	4.665250e-02		Jund	4.665250e-02	
de novo #104	23				Nr4a2	1.075660e-02		Nr4a3	1.075660e-02		Junb	1.846900e-01	
de novo #105	64				Jund	9.707080e-02		Hoxd11	9.707080e-02		Hoxd10	9.707080e-02	
de novo #106	122				Hoxd11	9.414860e-02		Hoxd10	9.414860e-02		Hoxc10	9.414860e-02	

pattern	num_seqlets	modisco_cwm_fwd	modisco_cwm_rev	delta_effects	match0	qval0	match0_logo	match1	qval1	match1_logo	match2	qval2	match2_logo
de novo #107	11581				Fosl2	2.181910e-03		Fos	2.181910e-03		Bach2	0.002871	
de novo #108	52				Mga	2.438900e-01		Tbx4	2.438900e-01		Tbx5	2.438900e-01	
de novo #109	23				Nr4a2	7.703990e-04		Nr4a3	7.703990e-04		Rarg	0.271699	
de novo #110	32				Mafa	6.076560e-03		Fosb	1.338000e-02		Bach2	4.036000e-02	
de novo #111	12835				Fosl2	2.152640e-03		Fos	2.152640e-03		Bach2	2.821790e-03	
de novo #112	500				Batf3	1.410160e-02		Foxb1	1.520540e-02		Foxb2	1.520540e-02	
de novo #113	6974				Fosl2	2.934750e-03		Fos	2.934750e-03		Bach2	3.051290e-03	
de novo #114	52				Nr1h3	3.141360e-03		Nr2c2	9.300220e-03		Pparg	9.300220e-03	
de novo #115	38				Hnf4g	5.211040e-03		Nr1h3	9.307750e-03		Hnf4a	3.346690e-02	
de novo #116	12534				Hnf4g	8.803960e-09		Hnf4a	1.308760e-05		Nr1h3	2.267470e-05	
de novo #117	39				Nr1h3	1.720710e-03		Nr2c2	1.720710e-03		Pparg	0.004057	
de novo #118	182				Hnf4g	1.587790e-08		Nr1h3	2.561520e-05		Hnf4a	0.000255	
de novo #119	83				Hnf4g	3.238640e-03		Rarg	9.019830e-03		Hnf4a	8.759430e-02	
de novo #120	27				Nr2c2	7.812650e-03		Ppard	7.812650e-03		Hnf4g	7.812650e-03	
de novo #121	11793				Hnf4g	1.486930e-07		Nr1h3	1.063210e-05		Hnf4a	1.063210e-05	

pattern	num_seqlets	modisco_cwm_fwd	modisco_cwm_rev	delta_effects	match0	qval0	match0_logo	match1	qval1	match1_logo	match2	qval2	match2_logo
de novo #122	7003				Hnf4g	2.734790e-08		Nr1h3	3.019100e-05		Hnf4a	3.128750e-05	
de novo #123	18200				Hnf4g	1.130930e-06		Hnf4a	1.626550e-05		Nr1h3	0.000022	
de novo #124	7794				Hnf4g	3.365370e-08		Nr1h3	2.324910e-05		Hnf4a	2.324910e-05	
de novo #125	59				Nr1h3	1.151940e-03		Nr1i2	1.151940e-03		Hnf4g	0.007071	
de novo #126	24734				Hnf4g	7.803890e-07		Nr1h3	8.006090e-06		Hnf4a	0.000020	
de novo #127	61				Ppard	4.537550e-04		Nr2c2	4.537550e-04		Hnf4g	2.768100e-03	
de novo #128	20574				Hnf4g	1.129920e-06		Nr1h3	1.801230e-05		Hnf4a	2.456620e-05	
de novo #129	30				Hnf4g	4.825680e-04		Nr1h3	3.379230e-03		Nr2c2	8.362710e-03	
de novo #130	53				Hnf4g	8.050360e-05		Hnf4a	5.189290e-03		Nr1h3	6.364160e-03	
de novo #131	43				Hnf4g	1.657000e-03		Nr1h3	3.191940e-03		Hnf4a	3.513060e-03	
de novo #132	48				Nr1h3	3.355830e-02		Sp2	3.355830e-02		Klf3	5.153700e-02	
de novo #133	56				Nr1h3	1.610720e-05		Hnf4g	5.836350e-04		Ppard	7.898150e-04	
de novo #134	99				Nr1h3	1.303280e-03		Ppard	1.303280e-03		Hnf4g	1.303280e-03	
de novo #135	21835				Hnf4g	3.216390e-07		Hnf4a	1.410670e-05		Nr1h3	1.410670e-05	
de novo #136	111				Hnf4a	4.482770e-06		Esrrb	5.794250e-02		Hnf4g	5.794250e-02	

pattern	num_seqlets	modisco_cwm_fwd	modisco_cwm_rev	delta_effects	match0	qval0	match0_logo	match1	qval1	match1_logo	match2	qval2	match2_logo
de novo #137	12297				Hnf4a	5.667580e-06		Hnf4g	5.667580e-06		Nr1h3	1.251150e-03	
de novo #138	46				Hnf4g	1.182750e-04		Ppard	1.244520e-04		Nr1h3	0.000553	
de novo #139	86				Hnf4a	4.470080e-06		Esrrb	5.697020e-02		Esrra	5.697020e-02	
de novo #140	23241				Hnf4g	2.367900e-06		Nr1h3	6.159820e-06		Hnf4a	2.403120e-05	
de novo #141	14406				Hnf4g	3.445030e-08		Hnf4a	1.550170e-05		Nr1h3	1.689000e-05	
de novo #142	9412				Hnf4g	2.774410e-08		Nr1h3	1.770000e-05		Hnf4a	1.770000e-05	
de novo #143	103				Hnf4g	2.330990e-04		Pparg	5.569710e-03		Nr2c2	5.569710e-03	
de novo #144	196				Nr5a1	2.828560e-03		Nr2c2	2.828560e-03		Esrra	2.828560e-03	
de novo #145	7289				Ppard	5.247990e-08		Nr2c2	3.908270e-06		Ppara	3.583600e-05	
de novo #146	41				Ppard	6.378380e-02		Nr2c2	9.832770e-02		Pparg	0.098328	
de novo #147	46				Mbtps2	4.365700e-02		Yy1	4.365700e-02		Yy2	4.365700e-02	
de novo #148	20020				Ctcf	3.987550e-18		Ctcfl	9.961500e-07		Zfp661	9.839710e-02	
de novo #149	19926				Ctcf	5.579200e-18		Ctcfl	8.469540e-07		Zfp661	1.004840e-01	
de novo #150	138				Ctcf	4.669660e-04		Ctcfl	1.091950e-02		Tcf12	3.923700e-02	
de novo #151	22787				Ctcf	2.172540e-18		Ctcfl	7.500890e-07		Zfp661	9.690380e-02	

pattern	num_seqlets	modisco_cwm_fwd	modisco_cwm_rev	delta_effects	match0	qval0	match0_logo	match1	qval1	match1_logo	match2	qval2	match2_logo
de novo #152	507				Ctcfl	9.343630e-06		Ctcf	2.663360e-05		Zfp46	2.595330e-01	
de novo #153	23263				Ctcf	3.508700e-18		Ctcfl	6.175850e-07		Zfp661	7.864470e-02	
de novo #154	21200				Ctcf	3.549170e-18		Ctcfl	9.396300e-07		Zfp661	8.634770e-02	
de novo #155	22073				Ctcf	9.767180e-19		Ctcfl	1.053800e-06		Zfp661	9.368690e-02	
de novo #156	21249				Ctcf	7.640640e-19		Ctcfl	5.911040e-07		Zfp661	1.022060e-01	
de novo #157	179				Ctcf	8.098070e-03		Ctcfl	2.508240e-02		Zfp423	1.000000e+00	
de novo #158	22155				Ctcf	2.045610e-18		Ctcfl	9.443880e-07		Zfp661	8.792090e-02	
de novo #159	555				Ctcfl	3.827220e-06		Ctcf	3.595430e-05		Zfp46	2.241990e-01	
de novo #160	721				Ctcf	3.066420e-05		Ctcfl	5.411670e-05		Zfp46	1.207990e-01	
de novo #161	20735				Ctcf	1.227970e-17		Ctcfl	5.000180e-07		Zfp661	0.092693	
de novo #162	1079				Ctcfl	1.064980e-06		Ctcf	2.694810e-06		Zfp661	1.507180e-01	
de novo #163	21440				Ctcf	2.227560e-17		Ctcfl	6.899160e-07		Zfp661	0.106021	
de novo #164	41				Ctcf	1.035510e-02		Mbtps2	2.563730e-02		Yy1	2.563730e-02	
de novo #165	122				Ctcfl	1.546560e-03		Ctcf	1.347320e-02		Klf11	0.017045	
de novo #166	80				Ctcf	8.216710e-03		Mbtps2	1.130980e-02		Yy1	1.130980e-02	

pattern	num_seqlets	modisco_cwm_fwd	modisco_cwm_rev	delta_effects	match0	qval0	match0_logo	match1	qval1	match1_logo	match2	qval2	match2_logo
de novo #167	22122				Ctcf	4.517090e-18		Ctcfl	6.391610e-07		Zfp661	8.821040e-02	
de novo #168	24				Ctcf	1.541540e-01		Neurod2	2.061180e-01		Rest	0.346957	
de novo #169	23303				Ctcf	3.663750e-18		Ctcfl	5.266890e-07		Zfp661	9.921580e-02	
de novo #170	4772				Foxi1	1.172530e-01		Nfyb	1.172530e-01		Nfya	1.172530e-01	
de novo #171	5682				Foxi1	1.289550e-01		Nfyb	1.289550e-01		Nfyc	1.289550e-01	
de novo #172	5606				Foxi1	1.270340e-01		Nfyb	1.270340e-01		Nfya	1.270340e-01	
de novo #173	5697				Foxi1	1.115870e-01		Nfyb	1.115870e-01		Nfya	0.111587	
de novo #174	4978				Foxi1	1.268460e-01		Nfyb	1.268460e-01		Nfyc	1.268460e-01	
de novo #175	5267				Foxi1	1.290340e-01		Nfyb	1.290340e-01		Nfyc	0.129034	
de novo #176	5021				Foxi1	1.004580e-01		Nfyb	1.004580e-01		Nfyc	1.004580e-01	
de novo #177	4803				Foxi1	1.268220e-01		Nfyb	1.268220e-01		Nfyc	1.268220e-01	
de novo #178	4863				Foxi1	1.134790e-01		Nfyb	1.134790e-01		Nfyc	1.134790e-01	
de novo #179	5453				Foxi1	1.275380e-01		Nfyb	1.275380e-01		Nfyc	1.275380e-01	
de novo #180	4833				Foxi1	5.700760e-02		Nfyb	5.700760e-02		Nfyc	5.700760e-02	
de novo #181	4808				Foxi1	1.266910e-01		Nfyb	1.266910e-01		Nfyc	1.266910e-01	

pattern	num_seqlets	modisco_cwm_fwd	modisco_cwm_rev	delta_effects	match0	qval0	match0_logo	match1	qval1	match1_logo	match2	qval2	match2_logo
de novo #182	59				Foxl2	5.489810e-03		Foxa1	1.098120e-02		Foxc1	1.098120e-02	
de novo #183	32				Foxa1	1.418390e-03		Foxc1	6.872920e-03		Foxd3	0.006873	
de novo #184	47				Foxa2	4.273560e-04		Foxa3	5.917090e-03		Foxa1	2.244270e-02	
de novo #185	151				Foxa1	3.325200e-03		Foxb1	3.325200e-03		Foxb2	3.325200e-03	
de novo #186	227				Foxb1	4.284640e-02		Foxb2	4.284640e-02		Foxa1	0.042846	
de novo #187	55				Foxa1	7.942870e-05		Foxb1	6.630460e-04		Foxb2	6.630460e-04	
de novo #188	60				Foxa1	4.094640e-01		Pou4f2	4.094640e-01		Foxb1	4.094640e-01	
de novo #189	609				Foxb1	1.197860e-01		Foxb2	1.197860e-01		Foxa1	1.197860e-01	
de novo #190	318				Batf3	2.248070e-02		Foxb1	2.248070e-02		Foxb2	2.248070e-02	
de novo #191	1200				Foxa1	3.555390e-04		Foxb1	3.555390e-04		Foxb2	3.555390e-04	
de novo #192	12015				Foxa1	2.896070e-05		Foxa3	2.896070e-05		Foxd1	4.545350e-05	
de novo #193	980				Foxa1	4.254500e-06		Foxb1	1.284130e-05		Foxb2	1.284130e-05	
de novo #194	716				Foxa1	8.324730e-04		Foxa2	1.310010e-03		Foxc1	1.310010e-03	
de novo #195	568				Foxa1	4.283670e-02		Foxb1	4.283670e-02		Foxb2	4.283670e-02	
de novo #196	198				Foxe1	2.070390e-03		Foxe3	2.070390e-03		Foxj1	0.007988	

pattern	num_seqlets	modisco_cwm_fwd	modisco_cwm_rev	delta_effects	match0	qval0	match0_logo	match1	qval1	match1_logo	match2	qval2	match2_logo
de novo #197	89				Foxa1	1.875180e-03		Foxd3	2.039810e-03		Foxa3	4.191990e-03	
de novo #198	2287				Foxa1	4.268350e-07		Foxb1	1.093420e-05		Foxb2	1.093420e-05	
de novo #199	2365				Foxa1	1.297570e-06		Foxb1	1.779460e-05		Foxb2	1.779460e-05	
de novo #200	5096				Foxa1	7.145400e-07		Foxa2	3.871600e-06		Foxa3	4.078610e-06	
de novo #201	283				Foxb1	3.966450e-02		Foxb2	3.966450e-02		Foxa1	3.966450e-02	
de novo #202	67				Foxe1	2.983470e-03		Foxe3	2.983470e-03		Foxa3	9.905380e-03	
de novo #203	12389				Foxa1	5.018210e-06		Foxa3	5.018210e-06		Foxd1	0.000005	
de novo #204	8789				Foxa1	1.157550e-06		Foxa3	2.315100e-06		Foxd1	2.700950e-06	
de novo #205	357				Foxe1	1.516140e-03		Foxe3	1.516140e-03		Foxj1	1.516140e-03	
de novo #206	5994				Foxa1	4.301150e-06		Foxa3	8.602300e-06		Foxd1	0.000010	
de novo #207	179				Foxa1	1.130600e-03		Foxb1	1.465900e-03		Foxb2	0.001466	
de novo #208	5420				Foxa1	8.615990e-06		Foxa3	8.615990e-06		Foxd1	8.615990e-06	
de novo #209	6463				Foxa1	2.479240e-07		Foxa3	1.738970e-06		Foxa2	1.738970e-06	
de novo #210	10097				Foxa1	1.354670e-05		Foxa3	1.354670e-05		Foxd1	1.354670e-05	
de novo #211	247				Foxe1	1.007770e-03		Foxe3	1.007770e-03		Foxj1	2.876580e-03	

pattern	num_seqlets	modisco_cwm_fwd	modisco_cwm_rev	delta_effects	match0	qval0	match0_logo	match1	qval1	match1_logo	match2	qval2	match2_logo
de novo #212	8419				Nfia	4.502530e-05		Nfix	1.257380e-04		Nfib	0.059880	
de novo #213	12038				Nfia	5.947000e-04		Nfix	1.557050e-03		Nfib	3.631430e-02	
de novo #214	10742				Nfia	2.474360e-04		Nfix	4.048930e-04		Nfib	5.993450e-02	
de novo #215	12936				Nfia	4.480720e-05		Nfix	5.994880e-05		Nfib	6.509320e-02	
de novo #216	12649				Nfia	3.252370e-04		Nfix	5.077820e-04		Nfib	0.057647	
de novo #217	12235				Nfia	5.496600e-05		Nfix	5.632610e-05		Nfib	7.937560e-02	
de novo #218	10021				Nfia	3.571630e-06		Nfix	1.253910e-05		Nfib	4.313630e-03	
de novo #219	17692				Nfia	2.047640e-04		Nfix	3.709800e-04		Nfib	3.466890e-03	
de novo #220	46				Nfia	1.239430e-04		Nfix	3.531860e-03		Nfic	0.283058	
de novo #221	8507				Nfia	1.132860e-05		Nfix	3.355680e-05		Nfib	3.203050e-03	
de novo #222	10327				Nfia	2.323610e-05		Nfix	2.323610e-05		Nfib	1.640720e-01	
de novo #223	148				Nfix	2.334390e-02		Nfia	2.334390e-02		Klf7	0.027380	
de novo #224	1970				Nfib	6.197450e-02		Nfia	6.197450e-02		Nfix	9.233940e-02	
de novo #225	10853				Nfia	1.765680e-05		Nfix	2.963110e-05		Nfib	1.269940e-01	
de novo #226	34				Nfix	1.719990e-02		Nfia	5.046160e-02		Insm1	5.212400e-02	

pattern	num_seqlets	modisco_cwm_fwd	modisco_cwm_rev	delta_effects	match0	qval0	match0_logo	match1	qval1	match1_logo	match2	qval2	match2_logo
de novo #227	10764				Nfia	3.399960e-05		Nfix	7.988520e-05		Nfib	7.969060e-02	
de novo #228	535				Batf3	1.201320e-01		Jund	5.808030e-01		Fosb	5.808030e-01	
de novo #229	405				Batf3	6.557990e-02		Fosb	1.519830e-01		Jund	2.326260e-01	
de novo #230	143				Elf3	1.518150e-01		Elf5	1.518150e-01		Elf1	1.518150e-01	
de novo #231	270				Ehf	1.300130e-04		Gabpa	1.887020e-04		Ets1	3.497400e-04	
de novo #232	45				Spic	3.344310e-01		Ets2	1.000000e+00		Ctcf	1.000000e+00	
de novo #233	168				Elf1	4.261680e-04		Ehf	6.256240e-04		Elf5	1.416600e-03	
de novo #234	253				Ehf	1.397990e-05		Elf5	7.486510e-04		Elf3	7.343490e-03	
de novo #235	35				Zfp296	7.098940e-01		Zkscan6	7.098940e-01		Ctcf	1.000000e+00	
de novo #236	180				Elk1	1.507500e-02		Elk4	1.507500e-02		Erg	1.507500e-02	
de novo #237	84				Elk1	3.330640e-02		Elk4	3.330640e-02		Elf1	9.536030e-02	
de novo #238	31				Ctcf	1.000000e+00		Hhex	1.000000e+00		Prdm16	1.000000e+00	
de novo #239	35				Dmrtc2	1.000000e+00		Rfx6	1.000000e+00		Prdm16	1.000000e+00	
de novo #240	951				Hoxb9	5.755880e-01		Cdx4	5.755880e-01		Hoxd10	5.755880e-01	
de novo #241	140				Hoxa13	2.975470e-01		Hoxd13	2.975470e-01		Hoxd9	3.253570e-01	

pattern	num_seqlets	modisco_cwm_fwd	modisco_cwm_rev	delta_effects	match0	qval0	match0_logo	match1	qval1	match1_logo	match2	qval2	match2_logo
de novo #242	234				Cdx1	5.725080e-05		Cdx2	5.725080e-05		Hoxd10	9.505580e-05	
de novo #243	439				Cdx2	1.547870e-03		Hoxa11	1.547870e-03		Hoxa10	1.600450e-03	
de novo #244	21438				Cdx2	1.928070e-04		Cdx4	7.573610e-04		Hoxb13	7.573610e-04	
de novo #245	275				Cdx2	1.263970e-04		Cdx1	6.821280e-04		Hoxa10	4.991530e-03	
de novo #246	37351				Cdx2	5.486950e-04		Hoxb13	5.690130e-04		Hoxd13	5.690130e-04	
de novo #247	35379				Cdx2	3.595990e-05		Cdx4	6.896940e-05		Hoxd10	1.585800e-04	
de novo #248	33178				Cdx2	2.812740e-04		Cdx4	9.083030e-04		Hoxb13	9.083030e-04	
de novo #249	31384				Cdx2	6.056690e-04		Cdx4	6.056690e-04		Hoxb13	0.000606	
de novo #250	36				Cdx2	6.982860e-02		Hoxd10	6.982860e-02		Cdx1	8.726180e-02	
de novo #251	16803				Cdx2	5.032580e-05		Cdx4	2.868030e-04		Hoxc9	2.536080e-03	
de novo #252	13651				Cdx2	1.741600e-04		Cdx4	7.255270e-04		Hoxb13	7.255270e-04	
de novo #253	111				Hoxd10	1.200030e-02		Cdx2	1.326890e-02		Cdx1	2.880910e-02	
de novo #254	51				Hoxd10	7.643280e-02		Cdx2	7.643280e-02		Cdx1	9.589870e-02	
de novo #255	110				Hoxd10	1.388310e-02		Cdx2	1.388310e-02		Cdx1	3.731710e-02	
de novo #256	262				Cdx1	5.384130e-06		Cdx2	2.597860e-04		Hoxa10	2.597860e-04	

pattern	num_seqlets	modisco_cwm_fwd	modisco_cwm_rev	delta_effects	match0	qval0	match0_logo	match1	qval1	match1_logo	match2	qval2	match2_logo
de novo #257	10539				Cdx2	1.347560e-03		Hoxb13	1.347560e-03		Hoxc13	1.347560e-03	
de novo #258	112				Dbx1	4.137620e-01		Hoxd10	4.137620e-01		Cdc5l	4.137620e-01	
de novo #259	89				Hoxd10	4.451880e-02		Cdx1	6.468280e-02		Hoxb8	0.064683	
de novo #260	8795				Cdx2	2.697980e-04		Cdx4	1.667080e-03		Hoxd13	1.993960e-03	
de novo #261	51				Cdx1	3.876740e-05		Hoxa11	1.214610e-04		Hoxa10	0.000148	
de novo #262	103				Dbx1	4.668750e-01		Zfp523	4.668750e-01		Cdc5l	4.668750e-01	
de novo #263	18363				Cdx2	3.721710e-04		Cdx4	1.599210e-03		Hoxb13	1.599210e-03	
de novo #264	310				Hoxa10	8.733650e-04		Hoxa11	8.733650e-04		Cdx2	8.733650e-04	
de novo #265	78				Dbx1	6.472410e-01		Onecut2	6.472410e-01		Cdc5l	0.647241	
de novo #266	124				Hoxd10	1.565380e-02		Cdx2	1.565380e-02		Cdx1	0.037209	
de novo #267	1352				Cdx4	5.856030e-01		Hoxc9	5.856030e-01		Hoxb9	5.856030e-01	
de novo #268	37925				Cdx2	4.964230e-05		Cdx4	1.478590e-04		Hoxd10	0.000161	
de novo #269	184				Hmg20a	9.583340e-02		Hmg20b	9.583340e-02		Hoxd10	1.796610e-01	
de novo #270	3467				Zbtb32	1.376540e-01		Cdx2	2.311090e-01		Hoxd10	5.991560e-01	
de novo #271	68				Cdx1	1.313970e-04		Cdx2	3.512360e-04		Hoxa10	3.512360e-04	

pattern	num_seqlets	modisco_cwm_fwd	modisco_cwm_rev	delta_effects	match0	qval0	match0_logo	match1	qval1	match1_logo	match2	qval2	match2_logo
de novo #272	2895				Zbtb32	1.469910e-01		Cdx2	1.469910e-01		Hoxa13	6.600880e-01	
de novo #273	157				Zfp653	1.000000e+00		Bsx	1.000000e+00		Hoxd9	1.000000	
de novo #274	299				Cdx1	2.974110e-05		Cdx2	3.717640e-05		Hoxd10	0.000098	
de novo #275	39407				Cdx2	6.096990e-04		Hoxb13	7.638510e-04		Hoxd13	7.638510e-04	
de novo #276	585				Sp2	6.319320e-05		Klf6	6.319320e-05		Sp3	0.000551	
de novo #277	84				Klf1	4.406910e-03		Klf4	6.206030e-03		Klf8	0.020599	
de novo #278	96				Klf6	7.676270e-04		Klf3	5.640010e-03		Patz1	5.640010e-03	
de novo #279	213				Sp3	1.252720e-03		Klf3	1.252720e-03		Sp5	1.743180e-03	
de novo #280	242				Klf3	5.943200e-08		Sp3	1.201580e-04		Klf7	1.418650e-04	
de novo #281	40567				Klf3	3.027440e-07		Klf12	5.907840e-05		Klf7	5.907840e-05	
de novo #282	387				Patz1	1.242280e-02		Klf2	4.117340e-02		Wt1	4.117340e-02	
de novo #283	71				Zfx	4.992440e-04		Klf6	4.992440e-04		Sp2	0.000499	
de novo #284	48189				Klf3	2.593820e-06		Klf4	3.570540e-05		Klf12	3.570540e-05	
de novo #285	182				Klf3	3.898810e-06		Klf12	3.898810e-06		Klf8	3.898810e-06	
de novo #286	32				Klf1	4.585310e-03		Klf4	4.585310e-03		Klf12	4.719290e-03	

pattern	num_seqlets	modisco_cwm_fwd	modisco_cwm_rev	delta_effects	match0	qval0	match0_logo	match1	qval1	match1_logo	match2	qval2	match2_logo
de novo #287	46808				Klf3	6.687110e-06		Klf4	6.687110e-06		Klf8	0.000009	
de novo #288	390				Klf3	7.962920e-07		Sp2	1.008760e-06		Sp3	2.495260e-05	
de novo #289	420				Patz1	1.168870e-02		Klf2	4.150580e-02		Wt1	4.150580e-02	
de novo #290	186				Patz1	6.237970e-03		Sp5	1.123540e-02		Wt1	5.220390e-02	
de novo #291	701				Klf3	1.796740e-04		Sp3	2.016780e-04		Sp2	5.998170e-04	
de novo #292	338				Klf12	5.998370e-04		Klf8	5.998370e-04		Klf7	5.998370e-04	
de novo #293	356				Klf12	7.138190e-04		Klf8	7.138190e-04		Klf7	0.000788	
de novo #294	342				Klf12	4.664650e-04		Klf8	4.664650e-04		Klf7	6.496850e-04	
de novo #295	47				Klf12	4.779050e-03		Klf8	4.779050e-03		Patz1	0.004779	
de novo #296	36				Klf6	3.000010e-03		Klf1	3.000010e-03		Klf10	3.000010e-03	
de novo #297	244				Sp2	9.364210e-04		Wt1	9.364210e-04		Klf15	9.364210e-04	
de novo #298	450				Sp2	3.083040e-04		Klf6	3.332050e-04		Klf15	0.000348	
de novo #299	1417				Klf3	1.506870e-06		Sp3	4.048320e-05		Klf6	3.412510e-04	
de novo #300	121				Klf6	5.740760e-04		Patz1	2.316560e-03		Klf3	6.776090e-03	
de novo #301	33				Klf2	2.087280e-02		Klf3	2.236360e-02		Klf12	2.469430e-02	

pattern	num_seqlets	modisco_cwm_fwd	modisco_cwm_rev	delta_effects	match0	qval0	match0_logo	match1	qval1	match1_logo	match2	qval2	match2_logo
de novo #302	296				Sp9	3.101230e-04		Sp2	6.149840e-04		Sp4	6.149840e-04	
de novo #303	399				Patz1	1.000620e-02		Klf2	3.195810e-02		Sp9	3.195810e-02	
de novo #304	87				Ehf	2.261150e-04		Sp2	1.514020e-03		Wt1	1.677400e-03	
de novo #305	145				Zfp692	8.529210e-03		Patz1	2.718220e-02		Sp2	2.718220e-02	
de novo #306	54788				Klf3	8.408220e-06		Klf4	1.161630e-05		Klf8	1.712320e-05	
de novo #307	104				Klf5	1.531390e-02		Klf4	2.329130e-02		Patz1	2.329130e-02	
de novo #308	59				Klf6	1.556330e-03		Klf3	4.837700e-03		Sp2	4.837700e-03	
de novo #309	95				Klf5	5.842840e-06		Klf4	5.842840e-06		Klf6	5.842840e-06	
de novo #310	130				Klf6	5.330380e-04		Patz1	2.173710e-03		Klf3	0.006323	
de novo #311	398				Patz1	1.449140e-02		Klf2	4.028890e-02		Sp9	4.028890e-02	
de novo #312	30				Sp2	5.337010e-05		Zfx	5.591200e-05		Sp3	9.218770e-04	
de novo #313	33142				Klf3	9.663900e-08		Klf7	1.717660e-04		Klf12	1.717660e-04	
de novo #314	30				Patz1	9.942720e-03		Elk4	2.835490e-02		Klf5	4.011260e-02	
de novo #315	142				Klf6	6.357090e-04		Patz1	3.308910e-03		Klf3	8.007750e-03	
de novo #316	156				Sp3	3.258880e-06		Klf3	2.031210e-05		Klf15	5.775400e-05	

pattern	num_seqlets	modisco_cwm_fwd	modisco_cwm_rev	delta_effects	match0	qval0	match0_logo	match1	qval1	match1_logo	match2	qval2	match2_logo
de novo #317	58030				Klf4	3.589340e-06		Klf3	8.746670e-06		Klf8	0.000009	
de novo #318	63				Zfp692	7.848610e-03		Klf3	1.426330e-02		Patz1	1.426330e-02	
de novo #319	100				Zfx	2.638370e-05		Sp2	3.084470e-03		Sp3	4.978180e-03	
de novo #320	243				Sp6	3.693430e-05		Klf14	6.686650e-04		Klf7	0.000669	
de novo #321	148				Sp3	1.946130e-03		Klf3	1.946130e-03		Patz1	1.946130e-03	
de novo #322	21				Klf1	3.362820e-03		Klf4	3.362820e-03		Klf12	4.805540e-03	
de novo #323	532				Patz1	1.137080e-02		Wt1	4.860800e-02		Klf2	4.860800e-02	
de novo #324	449				Klf3	6.303230e-05		Sp3	6.303230e-05		Sp2	1.742880e-04	
de novo #325	469				Patz1	8.489200e-03		Klf2	4.165850e-02		Wt1	0.041659	
de novo #326	218				Sp3	1.300020e-04		Zbtb17	1.300020e-04		Sp2	1.513900e-04	
de novo #327	410				Klf15	3.236060e-05		Wt1	3.236060e-05		Sp3	3.432940e-04	
de novo #328	100				Klf6	8.743660e-04		Klf3	5.966710e-03		Patz1	0.005967	
de novo #329	594				Klf3	3.733180e-05		Sp3	6.046500e-05		Sp2	2.037850e-04	
de novo #330	55021				Klf4	6.384850e-06		Klf3	9.681040e-06		Klf8	9.681040e-06	
de novo #331	47				Klf5	5.734230e-04		Klf8	5.734230e-04		Klf1	5.734230e-04	

pattern	num_seqlets	modisco_cwm_fwd	modisco_cwm_rev	delta_effects	match0	qval0	match0_logo	match1	qval1	match1_logo	match2	qval2	match2_logo
de novo #332	24				Nrf1	6.955650e-04		Klf12	2.876500e-02		Klf7	3.209170e-02	
de novo #333	235				Patz1	5.922350e-04		Sp3	5.922350e-04		Klf3	0.000852	
de novo #334	44793				Klf3	4.896940e-07		Klf6	2.441620e-05		Klf4	2.441620e-05	
de novo #335	540				Wt1	7.636360e-05		Klf15	7.636360e-05		Sp3	7.636360e-05	
de novo #336	449				Klf15	3.947330e-04		Sp3	3.947330e-04		Klf6	3.947330e-04	
de novo #337	36734				Klf3	4.489610e-07		Klf7	1.232920e-04		Klf12	1.232920e-04	
de novo #338	66				Klf6	1.244180e-03		Klf3	4.912110e-03		Sp2	5.818060e-03	
de novo #339	166				Patz1	6.176580e-04		Sp3	6.176580e-04		Klf3	7.126440e-04	
de novo #340	413				Patz1	1.403570e-02		Klf2	4.052360e-02		Sp9	4.052360e-02	
de novo #341	94				Klf6	9.827730e-04		Klf3	4.989960e-03		Patz1	4.989960e-03	
de novo #342	435				Sp3	1.130670e-04		Sp2	1.130670e-04		Klf10	1.078310e-03	
de novo #343	45935				Klf3	3.418860e-06		Klf4	1.837130e-05		Klf8	2.825550e-05	
de novo #344	371				Sp2	1.219880e-04		Sp3	1.219880e-04		Klf6	1.050030e-03	
de novo #345	245				Sp9	4.630510e-04		Sp4	6.761930e-04		Sp2	6.761930e-04	
de novo #346	169				Klf6	7.421430e-04		Wt1	7.421430e-04		Sp2	7.421430e-04	

pattern	num_seqlets	modisco_cwm_fwd	modisco_cwm_rev	delta_effects	match0	qval0	match0_logo	match1	qval1	match1_logo	match2	qval2	match2_logo
		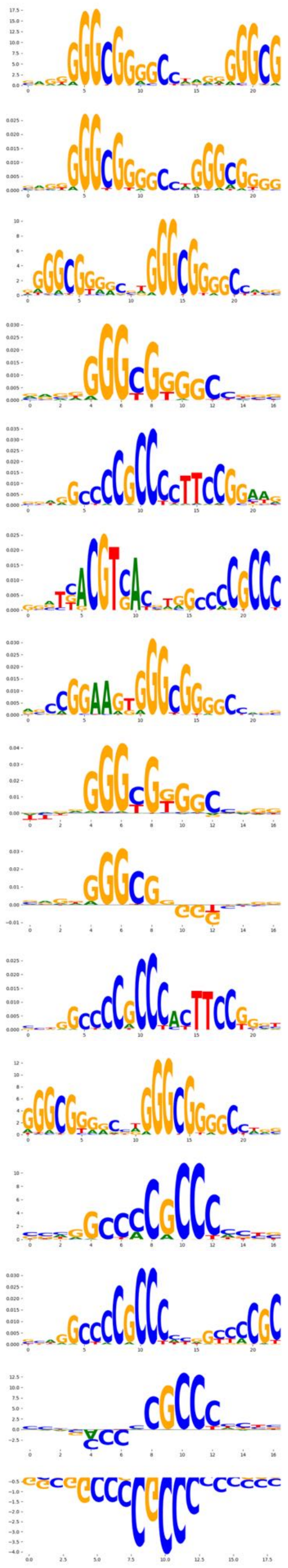	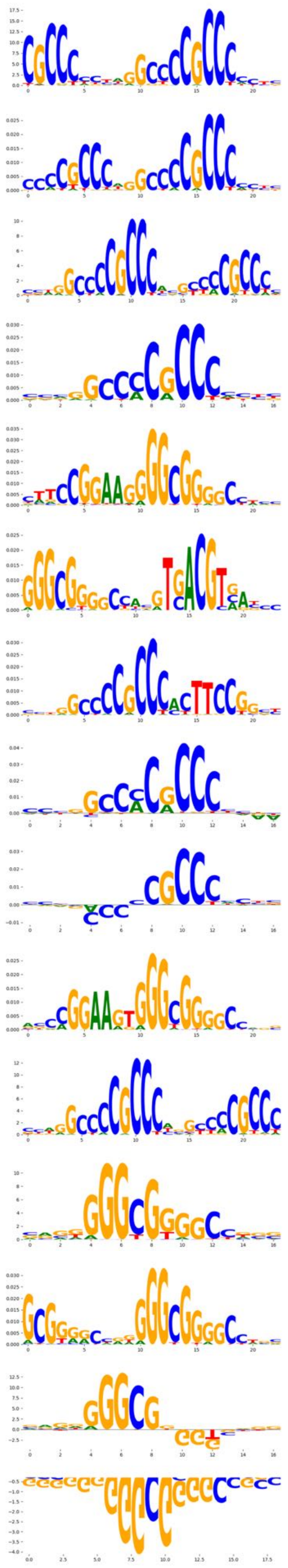	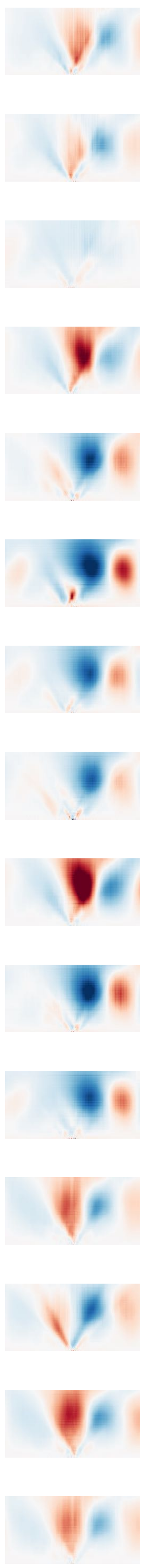			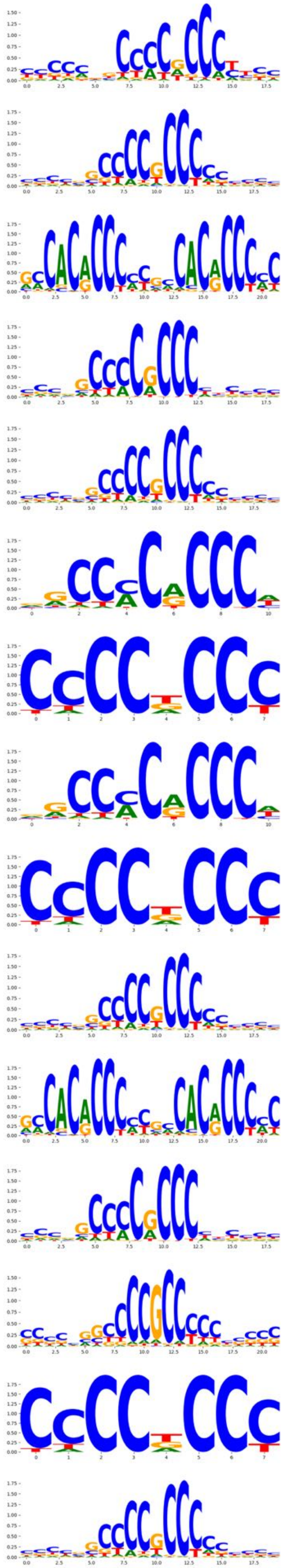			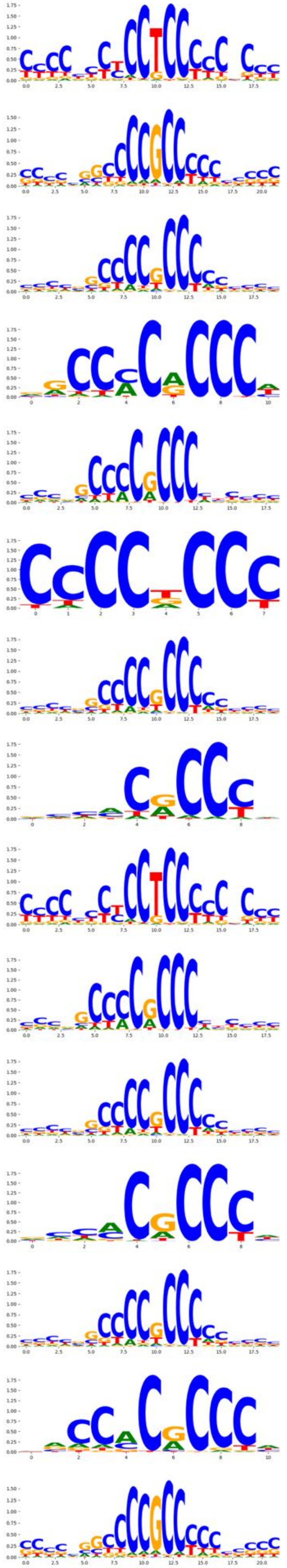			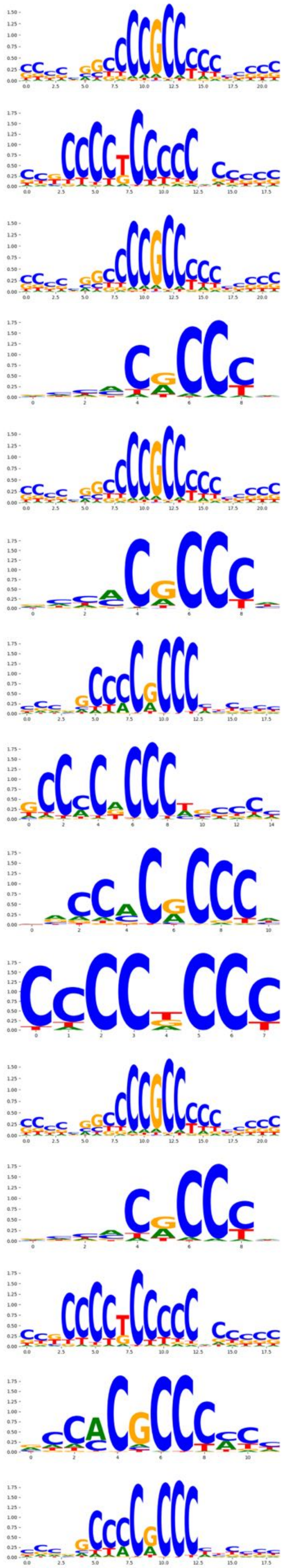
de novo #347	764				Sp3	8.996250e-05		Sp2	2.349200e-04		Zbtb17	0.000969	
de novo #348	428				Klf10	1.443460e-05		Sp3	1.443460e-05		Sp2	2.102810e-05	
de novo #349	52525				Klf3	5.610830e-06		Klf4	5.610830e-06		Klf8	7.988790e-06	
de novo #350	245				Sp3	1.996150e-03		Klf3	1.996150e-03		Sp2	1.996150e-03	
de novo #351	23				Klf4	9.555230e-03		Patz1	9.555230e-03		Klf7	1.289980e-02	
de novo #352	251				Patz1	1.469830e-04		Sp3	4.571180e-04		Klf3	5.614240e-04	
de novo #353	215				Klf4	3.044940e-06		Klf8	5.301790e-06		Klf5	0.000008	
de novo #354	479				Patz1	1.039740e-02		Wt1	4.570020e-02		Klf2	0.045700	
de novo #355	175				Sp3	2.013640e-03		Klf3	2.013640e-03		Patz1	2.013640e-03	
de novo #356	419				Klf10	1.660380e-05		Sp3	1.660380e-05		Sp2	1.898230e-05	
de novo #357	39601				Klf3	3.935730e-07		Klf7	7.105330e-05		Klf8	7.105330e-05	
de novo #358	631				Sp2	3.185080e-04		Sp3	4.855340e-04		Zbtb17	9.342300e-04	
de novo #359	437				Patz1	1.274330e-02		Klf2	3.531620e-02		Sp9	3.531620e-02	
de novo #360	1636				Sp3	3.243660e-07		Sp2	2.988540e-06		Klf3	1.354700e-04	
de novo #361	361				Sp3	3.123660e-04		Sp2	3.123660e-04		Klf15	3.123660e-04	

pattern	num_seqlets	modisco_cwm_fwd	modisco_cwm_rev	delta_effects	match0	qval0	match0_logo	match1	qval1	match1_logo	match2	qval2	match2_logo
de novo #362	69				Fosb	2.847850e-01		Hoxc5	2.847850e-01		Bsx	2.847850e-01	
de novo #363	7704				Pdx1	5.690690e-01		Arid3b	5.690690e-01		Otx2	5.690690e-01	
de novo #364	5932				Pdx1	5.586630e-01		Nanog	5.586630e-01		Hoxa4	0.558663	
de novo #365	4732				Pdx1	4.854910e-01		Arid3b	4.854910e-01		Nanog	4.854910e-01	
de novo #366	3483				Pdx1	5.476390e-01		Nanog	5.476390e-01		Hoxa4	5.476390e-01	
de novo #367	7278				Pdx1	5.526290e-01		Nanog	5.526290e-01		Arid3b	5.526290e-01	
de novo #368	7917				Pdx1	5.599420e-01		Hoxa4	5.599420e-01		Nanog	0.559942	
de novo #369	210				Hoxc10	1.695080e-01		Fosb	1.695080e-01		Hoxa4	1.695080e-01	
de novo #370	6445				Pdx1	5.397540e-01		Arid3b	5.397540e-01		Pou4f1	5.397540e-01	
de novo #371	7409				Pdx1	5.722250e-01		Nanog	5.722250e-01		Hoxa4	5.722250e-01	
de novo #372	4356				Pdx1	5.616770e-01		Nanog	5.616770e-01		Arid3b	5.616770e-01	
de novo #373	3683				Pdx1	5.483540e-01		Nanog	5.483540e-01		Arid3b	5.483540e-01	
de novo #374	227				Arid5a	8.572820e-01		Foxl2	8.572820e-01		Hmg20a	8.572820e-01	
de novo #375	6540				Pdx1	5.558850e-01		Nanog	5.558850e-01		Hoxa4	5.558850e-01	
de novo #376	5979				Pdx1	5.317380e-01		Nanog	5.317380e-01		Arid3b	5.317380e-01	

pattern	num_seqlets	modisco_cwm_fwd	modisco_cwm_rev	delta_effects	match0	qval0	match0_logo	match1	qval1	match1_logo	match2	qval2	match2_logo
de novo #377	87				Foxd2	8.650090e-01		Nanog	8.650090e-01		Sox15	0.865009	
de novo #378	138				Zik1	1.000000e+00		Zfp341	1.000000e+00		Bcl11a	1.000000e+00	
de novo #379	3110				Nfic	7.198460e-03		Nfib	4.216370e-02		Zbtb49	0.531475	
de novo #380	481				Klf7	3.325390e-01		Klf12	3.325390e-01		Klf8	3.325390e-01	
de novo #381	57				Klf1	4.075540e-02		Sp6	4.075540e-02		Klf7	4.075540e-02	
de novo #382	56				Klf1	4.190720e-02		Sp6	4.190720e-02		Klf7	4.190720e-02	
de novo #383	105				Sp2	4.702370e-05		Sp3	4.702370e-05		Wt1	7.348190e-04	
de novo #384	354				Klf7	5.726030e-01		Klf12	5.726030e-01		Klf8	5.726030e-01	
de novo #385	828				Klf7	5.209680e-01		Patz1	5.209680e-01		Klf12	5.209680e-01	
de novo #386	665				Klf7	4.182910e-01		Klf12	4.182910e-01		Klf8	4.182910e-01	
de novo #387	2297				Grhl2	1.251430e-06		Grhl1	1.543450e-05		Tfcp2	1.148610e-02	
de novo #388	26				Grhl2	9.361970e-03		Grhl1	2.466000e-02		Tfcp2	6.139840e-02	
de novo #389	1870				Grhl2	5.657740e-07		Grhl1	1.390640e-05		Tfcp2	1.129270e-02	
de novo #390	444				Grhl1	2.144290e-06		Grhl2	8.318640e-05		Tfcp2	1.182690e-02	
de novo #391	904				Grhl1	1.305210e-05		Grhl2	1.945310e-05		Tfcp2	1.154860e-02	

pattern	num_seqlets	modisco_cwm_fwd	modisco_cwm_rev	delta_effects	match0	qval0	match0_logo	match1	qval1	match1_logo	match2	qval2	match2_logo
de novo #392	897				Grhl2	8.416310e-06		Grhl1	8.416310e-06		Tfcp2	1.339060e-02	
de novo #393	3357				Grhl2	1.497430e-07		Grhl1	1.635860e-05		Tfcp2	0.012409	
de novo #394	943				Grhl2	1.854410e-05		Grhl1	1.854410e-05		Tfcp2	1.179520e-02	
de novo #395	1890				Grhl2	3.427030e-07		Grhl1	1.618990e-05		Tfcp2	1.157100e-02	
de novo #396	34				Grhl1	7.836940e-04		Fli1	1.602090e-01		Ets1	1.602090e-01	
de novo #397	2072				Grhl2	1.250760e-06		Grhl1	2.157480e-05		Tfcp2	1.403330e-02	
de novo #398	3335				Grhl2	8.281140e-07		Grhl1	1.900960e-05		Tfcp2	1.006200e-02	
de novo #399	298				Grhl1	1.026640e-03		Grhl2	1.026640e-03		Tfcp2	2.847210e-02	
de novo #400	1203				Grhl2	6.621740e-06		Grhl1	1.181850e-05		Tfcp2	0.012632	
de novo #401	93				Sox9	2.166990e-09		Sox3	9.277680e-03		Sox6	9.277680e-03	
de novo #402	161				Zkscan17	1.000000e+00		NaN	NaN		NaN	NaN	
de novo #403	310				Zbtb3	1.000000e+00		Ddit3	1.000000e+00		Atf4	1.000000e+00	
de novo #404	3745				Nrf1	3.821710e-07		Egr1	1.000000e+00		Egr4	1.000000e+00	
de novo #405	3776				Nrf1	4.693570e-06		Egr1	9.253110e-01		Mtf1	1.000000e+00	
de novo #406	4107				Nrf1	1.837240e-05		Egr1	8.938490e-01		Mtf1	8.938490e-01	

pattern	num_seqlets	modisco_cwm_fwd	modisco_cwm_rev	delta_effects	match0	qval0	match0_logo	match1	qval1	match1_logo	match2	qval2	match2_logo
de novo #407	3031				Nrf1	5.586510e-05		Hes7	9.702470e-01		Zfx	9.702470e-01	
de novo #408	70				Nrf1	2.351130e-02		Zfx	4.076190e-02		Sp2	0.232095	
de novo #409	27				Nrf1	9.136930e-03		Mnt	8.305800e-01		Myc	8.489310e-01	
de novo #410	2942				Nrf1	9.457550e-05		Hes7	9.323810e-01		Zfx	9.323810e-01	
de novo #411	3892				Nrf1	2.580790e-07		Egr1	1.000000e+00		Egr2	1.000000e+00	
de novo #412	2598				Nrf1	9.453140e-05		Zfx	8.468230e-01		Hes7	0.846823	
de novo #413	3216				Nrf1	5.593980e-05		Hes7	9.727930e-01		Zfx	0.972793	
de novo #414	3043				Nrf1	1.331300e-05		Egr1	8.532380e-01		Mtf1	1.000000e+00	
de novo #415	2686				Nrf1	1.642270e-05		Egr1	8.151840e-01		Mtf1	1.000000e+00	
de novo #416	4233				Nrf1	9.196980e-06		Mtf1	8.002040e-01		Egr1	8.002040e-01	
de novo #417	51				Zfp566	6.847360e-03		Nrf1	7.865330e-03		Zfp467	7.865330e-03	
de novo #418	46				Nrf1	1.539760e-03		Zfp467	1.539760e-03		Wt1	1.539760e-03	
de novo #419	55				Plagl1	7.459520e-03		Zfx	1.100360e-02		Nrf1	2.097070e-02	
de novo #420	3709				Nrf1	7.422720e-06		Egr1	9.324690e-01		Mtf1	1.000000e+00	
de novo #421	128				Nrf1	1.399850e-02		Mxd1	1.399850e-02		Mxi1	0.013999	

pattern	num_seqlets	modisco_cwm_fwd	modisco_cwm_rev	delta_effects	match0	qval0	match0_logo	match1	qval1	match1_logo	match2	qval2	match2_logo
de novo #422	1007				Foxd2	7.389820e-01		Foxg1	7.389820e-01		Foxi2	0.738982	
de novo #423	633				Foxd2	7.053940e-01		Foxg1	7.053940e-01		Foxi2	7.053940e-01	
de novo #424	640				Foxd2	7.752200e-01		Foxg1	7.752200e-01		Nkx61	7.752200e-01	
de novo #425	1069				Foxd2	7.763290e-01		Foxg1	7.763290e-01		Foxi2	7.763290e-01	
de novo #426	129				Foxd2	6.637840e-01		Arid5a	6.637840e-01		Nkx61	6.637840e-01	
de novo #427	10619				Ctcfl	5.226180e-02		Zfx	5.226180e-02		Sp2	5.226180e-02	
de novo #428	407				Sp2	1.156330e-08		Sp3	1.286370e-07		Klf3	1.518830e-04	
de novo #429	1684				Sp2	2.919030e-02		Sp3	2.919030e-02		Zfx	7.906170e-02	
de novo #430	192				Sp2	3.486450e-05		Sp3	3.486450e-05		Wt1	6.440840e-04	
de novo #431	1301				Zfx	2.648650e-04		Sp2	5.672660e-04		Zfp64	5.672660e-04	
de novo #432	1487				Sp2	6.418890e-08		Sp3	1.040180e-05		Zfx	1.251400e-04	
de novo #433	621				Sp2	2.650250e-03		Sp3	2.905590e-02		Zfx	0.029056	
de novo #434	331				Sp2	8.026460e-06		Sp3	1.509010e-05		Zfx	1.187160e-04	
de novo #435	955				Zbtb6	1.877220e-01		Mxd1	1.877220e-01		Mxi1	1.877220e-01	
de novo #436	259				Sp2	1.003340e-05		Sp3	3.522170e-05		Zfx	3.977780e-03	

pattern	num_seqlets	modisco_cwm_fwd	modisco_cwm_rev	delta_effects	match0	qval0	match0_logo	match1	qval1	match1_logo	match2	qval2	match2_logo
de novo #437	189				Sp2	1.837740e-06		Sp3	4.224710e-05		Zfx	3.714830e-04	
de novo #438	689				Sp2	7.069750e-02		Klf3	7.910440e-02		Sp3	0.079104	
de novo #439	33				Zfx	5.831020e-03		Sp2	5.831020e-03		Zfp64	0.005831	
de novo #440	516				Sp2	4.775550e-06		Sp3	3.418300e-05		Wt1	2.580020e-04	
de novo #441	104				Sp2	3.007390e-04		Zfx	1.882490e-03		Sp3	1.078100e-02	
de novo #442	67				Sp2	1.844670e-06		Sp3	6.892770e-06		Zfx	6.738980e-04	
de novo #443	115				Sp2	3.585010e-07		Sp3	7.071260e-06		Zfp566	3.171660e-04	
de novo #444	269				Sp2	7.158690e-07		Sp3	9.802470e-06		Zfx	1.055490e-03	
de novo #445	160				Sp2	6.546130e-05		Sp3	5.925930e-04		Klf3	2.847250e-03	
de novo #446	457				Sp2	2.003380e-06		Sp3	2.817990e-05		Wt1	1.773620e-03	
de novo #447	203				Sp2	7.898380e-07		Sp3	1.555440e-05		Klf3	1.182870e-03	
de novo #448	139				Zfp692	2.199180e-01		Zfp335	6.808590e-01		Zfp64	6.808590e-01	
de novo #449	51				Taf1	3.335580e-05		Mbtps2	3.777380e-05		Yy1	3.777380e-05	
de novo #450	472				Smad1	4.991070e-02		Smad5	4.991070e-02		Mbd2	6.723600e-02	
de novo #451	74				Sp2	2.378080e-04		Sp3	4.315520e-03		Zfx	7.060010e-03	

pattern	num_seqlets	modisco_cwm_fwd	modisco_cwm_rev	delta_effects	match0	qval0	match0_logo	match1	qval1	match1_logo	match2	qval2	match2_logo
de novo #452	29				Sp3	3.044650e-03		Zfp64	3.044650e-03		Zfx	3.561430e-03	
de novo #453	384				Sp2	1.537770e-07		Sp3	1.673940e-05		Zbtb17	3.229580e-04	
de novo #454	35				Sp2	8.278650e-04		Sp3	3.292050e-03		Zfx	3.292050e-03	
de novo #455	1624				Sp2	2.080270e-04		Zfx	3.827530e-04		Sp3	1.265280e-03	
de novo #456	240				Zfx	8.374740e-05		Sp2	2.329670e-04		Sp3	7.958590e-04	
de novo #457	1602				Sp2	3.238360e-08		Sp3	4.511210e-06		Zfp566	7.587850e-04	
de novo #458	1086				Sp2	5.507110e-05		Sp3	1.269890e-03		Zfx	1.675190e-03	
de novo #459	24				Sp2	1.902450e-05		Sp3	4.164660e-04		Zfx	1.019300e-03	
de novo #460	540				Sp2	1.205750e-05		Sp3	3.276170e-05		Zfx	1.133950e-04	
de novo #461	311				Sp2	2.510890e-05		Sp3	4.483830e-05		Wt1	2.742480e-04	
de novo #462	4912				Zfx	2.282850e-03		Sp2	2.282850e-03		Sp3	8.065960e-03	
de novo #463	351				Sp2	6.917690e-07		Sp3	5.542800e-06		Zbtb17	1.008880e-04	
de novo #464	807				Sp2	1.617090e-04		Zfx	1.617090e-04		Sp3	5.658190e-04	
de novo #465	160				Sp2	1.130950e-04		Zfx	7.205940e-04		Sp3	7.205940e-04	
de novo #466	2256				Sp3	1.864920e-04		Sp2	1.864920e-04		Zfx	4.974510e-03	

pattern	num_seqlets	modisco_cwm_fwd	modisco_cwm_rev	delta_effects	match0	qval0	match0_logo	match1	qval1	match1_logo	match2	qval2	match2_logo
de novo #467	2909				Sp2	1.211360e-03		Ctcfl	1.211360e-03		Zfx	1.211360e-03	
de novo #468	490				Sp2	2.815930e-03		Zfx	2.815930e-03		Sp3	6.204260e-03	
de novo #469	7659				Sp2	4.800970e-07		Sp3	1.110760e-05		Zfx	1.639090e-04	
de novo #470	519				Sp2	9.048350e-05		Sp3	1.029990e-04		Zfp64	2.791350e-04	
de novo #471	401				Sp2	4.525810e-06		Sp3	9.712550e-05		Klf3	3.579060e-03	
de novo #472	119				Creb3	4.107630e-02		Gm28047	6.015100e-02		Sp3	6.015100e-02	
de novo #473	309				Myc	2.893450e-02		Sp3	2.893450e-02		Sp2	2.893450e-02	
de novo #474	888				Sp2	6.290030e-02		Mbd2	6.290030e-02		Klf3	6.290030e-02	
de novo #475	1839				Mbd2	1.447300e-01		Sp2	1.447300e-01		Smad1	0.144730	
de novo #476	35				Sp2	5.155330e-05		Sp3	4.811090e-04		Zfx	4.811090e-04	
de novo #477	552				Nrf1	4.047090e-01		Tfdp1	4.047090e-01		Tfdp2	4.047090e-01	
de novo #478	308				Sp2	1.078270e-04		Sp3	4.135760e-04		Wt1	4.751010e-04	
de novo #479	1770				Sp2	2.207820e-02		Zbtb6	8.800450e-02		Nrf1	0.088004	
de novo #480	2569				Sp2	1.149380e-04		Zfp566	1.336010e-03		Sp3	1.336010e-03	
de novo #481	656				Sp2	1.037480e-06		Sp3	1.157920e-05		Wt1	8.850050e-05	

pattern	num_seqlets	modisco_cwm_fwd	modisco_cwm_rev	delta_effects	match0	qval0	match0_logo	match1	qval1	match1_logo	match2	qval2	match2_logo
de novo #482	1210				Zbtb14	3.371190e-01		Nrf1	3.371190e-01		Hes1	3.371190e-01	
de novo #483	548				Sp2	8.628960e-04		Zfx	8.628960e-04		Sp3	1.330830e-03	
de novo #484	416				Sp3	7.836470e-07		Sp2	1.142720e-06		Zfp341	2.238730e-05	
de novo #485	587				Sp2	1.660750e-07		Sp3	3.810260e-07		Wt1	1.227350e-04	
de novo #486	118				Sp2	1.500640e-05		Sp3	1.418300e-04		Zfx	1.906300e-03	
de novo #487	17465				Sp2	1.048780e-03		Zfx	1.048780e-03		Sp3	4.453680e-03	
de novo #488	4867				Zfx	6.824050e-03		Sp2	6.824050e-03		Mxd1	6.824050e-03	
de novo #489	28				Sp2	2.940820e-04		Sp3	3.188680e-04		Zfx	1.501660e-03	
de novo #490	3741				Sp2	9.483770e-06		Sp3	4.639050e-04		Zfx	1.465260e-03	
de novo #491	2276				Sp2	3.660480e-08		Sp3	3.569200e-06		Zfx	2.023900e-04	
de novo #492	72				Zfx	9.778580e-02		Zbtb14	9.778580e-02		Hes1	9.778580e-02	
de novo #493	3741				Mbd2	1.043410e-01		Sp2	1.043410e-01		Dnmt1	1.043410e-01	
de novo #494	81				Taf1	2.038650e-04		Mbtps2	2.760060e-04		Yy1	2.760060e-04	
de novo #495	2226				Sp2	2.199360e-01		Sp3	2.199360e-01		Klf3	2.199360e-01	
de novo #496	1774				Mbd2	1.265820e-01		Smad1	1.265820e-01		Smad5	1.265820e-01	

pattern	num_seqlets	modisco_cwm_fwd	modisco_cwm_rev	delta_effects	match0	qval0	match0_logo	match1	qval1	match1_logo	match2	qval2	match2_logo
de novo #497	838				Sp2	1.889550e-03		Sp3	2.815070e-03		Zfx	2.815070e-03	
de novo #498	1263				Zbtb14	3.540240e-01		Nrf1	3.540240e-01		Myc	3.540240e-01	
de novo #499	654				Sp2	1.023700e-04		Zfx	1.861820e-04		Sp3	1.241750e-03	
de novo #500	288				Taf1	3.593070e-04		Mbtps2	1.224330e-03		Yy1	1.224330e-03	
de novo #501	1434				Sp2	4.411280e-04		Zfx	1.968590e-03		Sp3	2.261570e-03	
de novo #502	323				Sp2	7.996990e-07		Sp3	1.824870e-05		Zfx	2.032780e-04	
de novo #503	1154				Sp2	2.478820e-07		Sp3	1.610370e-05		Zfp566	8.610550e-04	
de novo #504	243				Sp2	1.890070e-04		Sp3	6.242990e-04		Zfp467	1.196090e-03	
de novo #505	19510				Sp2	2.215670e-03		Zfx	3.720200e-03		Zfp64	3.720200e-03	
de novo #506	2237				Zfx	7.074940e-05		Sp2	5.815480e-04		Sp3	3.022840e-03	
de novo #507	508				Taf1	2.155860e-02		Smad1	2.155860e-02		Smad5	2.155860e-02	
de novo #508	112				Sp2	1.981210e-06		Zfx	1.947790e-04		Sp3	1.947790e-04	
de novo #509	1543				Sp2	3.472400e-05		Sp3	4.015850e-04		Zfx	4.256450e-04	
de novo #510	274				Sp2	6.912760e-07		Sp3	6.912760e-07		Zfp341	4.454340e-05	
de novo #511	1889				Sp2	1.682040e-05		Zfx	1.101120e-04		Sp3	1.664410e-04	

pattern	num_seqlets	modisco_cwm_fwd	modisco_cwm_rev	delta_effects	match0	qval0	match0_logo	match1	qval1	match1_logo	match2	qval2	match2_logo
de novo #512	3988				Sp2	7.910310e-02		Dnmt1	7.910310e-02		Mxd1	7.910310e-02	
de novo #513	33				Sp2	8.390720e-05		Sp3	3.997870e-03		Wt1	1.344560e-02	
de novo #514	26				Sp2	5.556310e-05		Sp3	2.540460e-03		Elk4	3.379820e-03	
de novo #515	17777				Zfx	3.105580e-04		Sp2	2.345680e-03		Zfp64	7.655260e-03	
de novo #516	266				Sp2	2.935220e-05		Sp3	5.782690e-05		Maz	2.626850e-04	
de novo #517	55				Sp2	2.800000e-03		Zfx	2.800000e-03		Sp3	2.800000e-03	
de novo #518	3694				Zfx	1.633070e-04		Sp2	8.041980e-04		Mxd1	3.713290e-03	
de novo #519	53				Sp2	7.344460e-07		Sp3	5.514750e-06		Zfp566	9.358760e-04	
de novo #520	502				Sp2	3.895490e-07		Sp3	1.178000e-05		Zbtb17	4.681950e-04	
de novo #521	430				Sp2	1.427090e-06		Sp3	1.438200e-06		Zfp341	3.641010e-05	
de novo #522	7052				Sp2	1.787360e-10		Sp3	1.693940e-07		Zfx	2.540330e-05	
de novo #523	1505				Tfdp1	2.140370e-01		Tfdp2	2.140370e-01		Ctcfl	3.052060e-01	
de novo #524	137				Sp3	7.528860e-05		Sp2	9.980750e-05		Zfx	1.830400e-04	
de novo #525	128				Sp2	6.717960e-04		Sp3	3.291530e-03		Zfp64	6.098970e-03	
de novo #526	230				Taf1	5.340220e-02		Zfx	5.340220e-02		Sp3	5.340220e-02	

pattern	num_seqlets	modisco_cwm_fwd	modisco_cwm_rev	delta_effects	match0	qval0	match0_logo	match1	qval1	match1_logo	match2	qval2	match2_logo
de novo #527	29				Sp2	1.134230e-04		Sp3	1.680310e-04		Klf6	2.881910e-03	
de novo #528	303				Sp2	2.523590e-06		Sp3	3.201020e-06		Zfx	1.651990e-04	
de novo #529	685				Sp2	1.592250e-03		Sp3	2.901650e-03		Ctcfl	5.621050e-03	
de novo #530	1223				Zfx	2.123270e-02		Sp2	2.708080e-02		Sp3	8.156360e-02	
de novo #531	1579				Dlx5	1.864160e-02		Dlx2	1.864160e-02		Dlx4	1.864160e-02	
de novo #532	2316				Dlx5	2.127220e-02		Dlx2	2.127220e-02		Dlx4	2.127220e-02	
de novo #533	580				Nkx62	7.312600e-03		Dlx2	7.312600e-03		Hoxb7	0.007313	
de novo #534	2401				Dlx2	1.187790e-02		Dlx5	1.187790e-02		Dlx4	0.012772	
de novo #535	1496				Dlx5	2.614600e-02		Dlx2	2.614600e-02		Dlx4	2.614600e-02	
de novo #536	1307				Dlx5	2.105110e-02		Dlx2	2.105110e-02		Dlx4	2.105110e-02	
de novo #537	879				Zeb2	1.214930e-01		Figla	1.214930e-01		Tcf12	1.214930e-01	
de novo #538	65				Pparg	9.389520e-03		Nr2c2	4.297410e-02		Nr1h3	1.713410e-01	
de novo #539	16606				Erg	1.825100e-04		Elf5	2.429310e-03		Ehf	2.658150e-03	
de novo #540	108				Etv2	7.552730e-03		Fli1	7.552730e-03		Etv3	7.552730e-03	
de novo #541	9111				Elf2	1.807040e-05		Elf4	1.807040e-05		Erg	1.807040e-05	

pattern	num_seqlets	modisco_cwm_fwd	modisco_cwm_rev	delta_effects	match0	qval0	match0_logo	match1	qval1	match1_logo	match2	qval2	match2_logo
de novo #542	20716				Erg	3.718530e-03		Elf5	1.149950e-02		Ehf	0.023087	
de novo #543	50				Elk1	3.637570e-04		Erg	7.149790e-04		Erf	8.550150e-04	
de novo #544	13932				Erg	3.819040e-06		Ehf	1.678830e-04		Elf2	0.000168	
de novo #545	22				Elf5	4.744610e-05		Ehf	2.369810e-04		Elf3	5.471440e-04	
de novo #546	40				Elk1	4.606100e-03		Erf	5.121080e-03		ENSMUSG00000098546	5.121080e-03	
de novo #547	12191				Erg	7.482000e-06		Elf4	1.705560e-04		Ehf	1.705560e-04	
de novo #548	12601				Erg	3.080990e-05		Elf4	1.011380e-04		Ehf	1.430920e-04	
de novo #549	14174				Erg	7.557410e-06		Ehf	2.110140e-04		Elf4	2.110140e-04	
de novo #550	162				Spib	5.994760e-03		Bcl11a	6.983680e-03		Elk1	6.983680e-03	
de novo #551	188				Elk1	9.170160e-03		Spib	9.170160e-03		Erg	9.170160e-03	
de novo #552	18907				Erg	4.224970e-04		Elf5	2.142280e-03		Ehf	2.675360e-03	
de novo #553	12059				Erg	4.396570e-06		Elf2	2.105120e-04		Elf4	2.105120e-04	
de novo #554	73				Ehf	4.165400e-04		Elf5	4.348800e-04		Elf3	4.505640e-03	
de novo #555	10146				Erg	1.579100e-05		Elf2	1.579100e-05		Elf4	1.579100e-05	
de novo #556	23				Etv5	1.569820e-02		Ehf	1.569820e-02		Elk1	1.569820e-02	

pattern	num_seqlets	modisco_cwm_fwd	modisco_cwm_rev	delta_effects	match0	qval0	match0_logo	match1	qval1	match1_logo	match2	qval2	match2_logo
de novo #557	21219				Erg	4.005710e-03		Elf5	1.026660e-02		Ehf	1.796820e-02	
de novo #558	14248				Erg	2.698990e-06		Elf4	2.607810e-04		Ehf	2.607810e-04	
de novo #559	1372				Klf11	2.545080e-03		Sp1	2.545080e-03		Sp4	0.002545	
de novo #560	215				Zfp46	1.177460e-01		Klf1	2.472850e-01		Mga	5.662640e-01	
de novo #561	502				Ovol1	8.461450e-01		Zfp523	8.461450e-01		Nfe2l2	8.461450e-01	
de novo #562	610				Ovol1	8.743530e-01		Zfp523	8.743530e-01		Nfe2l2	8.743530e-01	
de novo #563	673				Ovol1	1.000000e+00		Nfe2l2	1.000000e+00		Fosl2	1.000000e+00	
de novo #564	254				Zfp46	9.215100e-02		Klf1	2.408690e-01		Mga	5.244020e-01	
de novo #565	529				Ovol1	9.841460e-01		Nfe2l2	9.841460e-01		Fosl2	9.841460e-01	
de novo #566	546				Ovol1	9.541000e-01		Nfe2l2	9.541000e-01		Fosl2	9.541000e-01	
de novo #567	591				Ovol1	1.000000e+00		Nfe2l2	1.000000e+00		Fosl2	1.000000e+00	
de novo #568	513				Ovol1	9.996910e-01		Nfe2l2	9.996910e-01		Fosl2	9.996910e-01	
de novo #569	499				Ovol1	7.336430e-01		Zfp523	7.336430e-01		Fosl2	0.733643	
de novo #570	578				Ovol1	1.000000e+00		Fosl2	1.000000e+00		Nfe2l2	1.000000e+00	
de novo #571	742				Ovol1	1.000000e+00		Fosl2	1.000000e+00		Nfe2l2	1.000000000	

pattern	num_seqlets	modisco_cwm_fwd	modisco_cwm_rev	delta_effects	match0	qval0	match0_logo	match1	qval1	match1_logo	match2	qval2	match2_logo
de novo #572	556				Ovol1	9.473760e-01		Zfp523	9.473760e-01		Fosl2	9.473760e-01	
de novo #573	27				Twist2	1.000000e+00		Zbtb18	1.000000e+00		Elk1	1.000000e+00	
de novo #574	43				Dmrtc2	9.606400e-01		Hoxb9	9.606400e-01		Lcorl	9.606400e-01	
de novo #575	350				Zfp335	1.000000e+00		Rela	1.000000e+00		Ovol1	1.000000e+00	
de novo #576	641				Hoxc6	1.315880e-02		Hoxb1	1.962610e-02		Nkx62	1.962610e-02	
de novo #577	1978				Zbtb7a	1.206780e-03		Glis2	6.915580e-02		Hnf4a	6.915580e-02	
de novo #578	2007				Zbtb7a	8.009020e-02		Glis2	8.009020e-02		Hnf4a	0.080090	
de novo #579	121				Glis3	2.471620e-02		Zfp641	2.471620e-02		Glis1	2.471620e-02	
de novo #580	49				Plagl1	2.807300e-02		Zfp64	1.275470e-01		Zbtb7a	1.883630e-01	
de novo #581	3174				Glis2	2.232460e-02		Zbtb7a	2.232460e-02		Zbtb7b	8.065880e-02	
de novo #582	3915				Hnf4a	7.196640e-02		Zbtb7a	7.196640e-02		Glis2	7.196640e-02	
de novo #583	66				Maz	1.438880e-04		Sp2	1.452870e-04		Sp3	0.000199	
de novo #584	168				Zfp64	2.200920e-02		Zbtb7a	2.918490e-02		Prdm4	1.231570e-01	
de novo #585	1613				Hnf4a	7.736430e-02		Glis2	7.736430e-02		Zbtb7a	7.736430e-02	
de novo #586	2077				Zbtb7a	7.627620e-04		Hnf4a	5.237320e-02		Glis1	0.068319	

pattern	num_seqlets	modisco_cwm_fwd	modisco_cwm_rev	delta_effects	match0	qval0	match0_logo	match1	qval1	match1_logo	match2	qval2	match2_logo
de novo #587	4283				Zbtb7a	2.929870e-02		Glis2	5.245800e-02		Glis1	5.245800e-02	
de novo #588	2585				Zbtb7a	2.692340e-03		Zbtb7b	4.919140e-02		Glis1	5.804950e-02	
de novo #589	2688				Zbtb7a	8.071270e-04		Zbtb7b	2.359110e-02		Glis2	6.916950e-02	
de novo #590	102				Zfp641	4.606480e-02		Glis3	4.606480e-02		Glis1	4.606480e-02	
de novo #591	5606				Zbtb7a	3.106770e-02		Glis2	5.588290e-02		Glis1	5.588290e-02	
de novo #592	4280				Glis2	1.631740e-02		Zbtb7a	1.631740e-02		Zbtb7b	9.336160e-02	
de novo #593	3593				Zbtb7a	7.458370e-04		Glis2	6.840810e-02		Zbtb7b	6.840810e-02	
de novo #594	1933				Glis2	2.620250e-02		Zbtb7a	3.881970e-02		Glis1	1.541390e-01	
de novo #595	3498				Zbtb7a	4.505360e-02		Glis2	4.505360e-02		Glis1	4.505360e-02	
de novo #596	2296				Maz	6.902630e-08		Zfp467	6.902630e-08		Sp5	6.902630e-08	
de novo #597	28				Zfp467	5.417810e-07		Maz	5.417810e-07		Sp3	1.006690e-06	
de novo #598	48				Maz	3.952810e-03		Zfp467	3.952810e-03		Sp2	4.572040e-03	
de novo #599	46				Sp5	4.976320e-07		Maz	4.976320e-07		Sp2	4.976320e-07	
de novo #600	350				Sp2	2.551980e-08		Zfp467	4.939670e-08		Maz	4.939670e-08	
de novo #601	1468				Sp5	7.040880e-06		Maz	1.059470e-05		Zfp467	1.120480e-05	

pattern	num_seqlets	modisco_cwm_fwd	modisco_cwm_rev	delta_effects	match0	qval0	match0_logo	match1	qval1	match1_logo	match2	qval2	match2_logo
de novo #602	1866				Sp5	3.394130e-08		Maz	5.456360e-08		Zfp467	5.456360e-08	
de novo #603	1757				Maz	8.503750e-08		Zfp467	8.503750e-08		Sp5	1.052670e-07	
de novo #604	843				Zfp341	2.290070e-06		Maz	6.308190e-06		Zfp467	6.308190e-06	
de novo #605	1347				Maz	4.966840e-08		Zfp467	4.966840e-08		Sp5	2.744210e-07	
de novo #606	115				Maz	9.437750e-05		Sp2	2.892090e-04		Klf15	2.892090e-04	
de novo #607	97				Klf15	3.291430e-04		Sp2	3.291430e-04		Maz	3.291430e-04	
de novo #608	80				Sp2	6.545750e-03		Maz	6.545750e-03		Sp3	0.006546	
de novo #609	250				Sp5	6.668320e-07		Maz	6.668320e-07		Zfp467	6.668320e-07	
de novo #610	1361				Sp3	8.639400e-07		Maz	8.639400e-07		Zfp467	9.226630e-07	
de novo #611	70				Sp5	8.193750e-05		Zfp281	8.193750e-05		Maz	0.000082	
de novo #612	33				Patz1	2.342000e-03		Wt1	3.477450e-03		Zbtb17	3.477450e-03	
de novo #613	31				Sp2	1.176730e-04		Sp3	1.176730e-04		Wt1	2.576530e-03	
de novo #614	213				Zfp64	5.893790e-03		Sp3	5.893790e-03		Sp2	0.005894	
de novo #615	557				Nfkb1	1.019130e-05		Rela	8.694490e-05		Relb	2.314570e-04	
de novo #616	342				Nfkb1	6.066220e-06		Rela	4.763520e-05		Relb	1.919460e-04	

pattern	num_seqlets	modisco_cwm_fwd	modisco_cwm_rev	delta_effects	match0	qval0	match0_logo	match1	qval1	match1_logo	match2	qval2	match2_logo
de novo #617	232				Nfkb1	2.616990e-06		Rela	2.982920e-05		Relb	1.199840e-04	
de novo #618	1213				Nfkb1	2.825430e-07		Rela	2.496670e-05		Nfkb2	4.563870e-04	
de novo #619	1683				Nfkb1	2.971170e-08		Rela	1.518700e-05		Nfkb2	0.000159	
de novo #620	517				Nfkb1	1.228650e-06		Rela	1.427660e-04		Nfkb2	6.350990e-04	
de novo #621	34				Nfkb1	1.815330e-05		Rela	1.965980e-04		Nfkb2	1.228590e-03	
de novo #622	669				Nfkb1	2.725840e-09		Rela	1.716040e-05		Relb	2.145970e-04	
de novo #623	62				Nfkb1	1.513140e-06		Rela	2.027780e-05		Relb	2.215010e-04	
de novo #624	241				Nfkb1	4.418140e-06		Rela	3.402780e-05		Relb	0.000325	
de novo #625	1162				Elf5	2.568690e-01		Elf3	2.568690e-01		Spi1	0.256869	
de novo #626	1019				Elf5	6.028360e-01		Elf3	6.028360e-01		Spi1	6.539720e-01	
de novo #627	1431				Elf5	7.716080e-01		Elf3	7.716080e-01		Spi1	0.771608	
de novo #628	2400				Elf5	1.566210e-01		Elf3	1.566210e-01		Spi1	1.939480e-01	
de novo #629	1275				Elf5	2.220800e-01		Elf3	2.220800e-01		Spi1	2.527070e-01	
de novo #630	23				Zfp212	8.411510e-01		Rreb1	9.075840e-01		Egr2	1.000000e+00	
de novo #631	2943				Rreb1	1.000000e+00		T	1.000000e+00		Egr2	1.000000e+00	

pattern	num_seqlets	modisco_cwm_fwd	modisco_cwm_rev	delta_effects	match0	qval0	match0_logo	match1	qval1	match1_logo	match2	qval2	match2_logo
de novo #632	1357				Rreb1	1.000000e+00		T	1.000000e+00		Egr2	1.000000e+00	
de novo #633	184				Klf1	2.327640e-01		Egr2	2.327640e-01		Rreb1	2.327640e-01	
de novo #634	814				Rreb1	1.000000e+00		T	1.000000e+00		Egr2	1.000000e+00	
de novo #635	3408				Rreb1	1.000000e+00		T	1.000000e+00		Egr2	1.000000e+00	
de novo #636	176				Egr2	1.229960e-02		Rreb1	1.229960e-02		Klf1	9.907460e-02	
de novo #637	1236				Rreb1	1.000000e+00		T	1.000000e+00		Egr2	1.000000e+00	
de novo #638	2995				Rreb1	1.000000e+00		T	1.000000e+00		Egr2	1.000000e+00	
de novo #639	126				Egr2	1.779170e-01		Mxd1	1.779170e-01		Mxi1	1.779170e-01	
de novo #640	65				Zfp661	2.019660e-03		Ctcfl	2.134500e-02		Ctcf	4.519710e-02	
de novo #641	63				Zfp661	2.115260e-03		Ctcfl	1.811420e-02		Ctcf	3.817170e-02	
de novo #642	51				Zfp661	1.630130e-03		Ctcfl	1.187340e-02		Ctcf	2.978670e-02	
de novo #643	263				Ctcf	1.000000e+00		Ctcfl	1.000000e+00		Plag1	1.000000e+00	
de novo #644	64				Zfp661	1.617330e-03		Ctcfl	1.681910e-02		Ctcf	3.777740e-02	
de novo #645	357				Ctcf	6.307190e-01		Ctcfl	9.299740e-01		Klf13	1.000000e+00	
de novo #646	183				Ctcf	7.451990e-01		Ctcfl	7.451990e-01		Plag1	1.000000e+00	

pattern	num_seqlets	modisco_cwm_fwd	modisco_cwm_rev	delta_effects	match0	qval0	match0_logo	match1	qval1	match1_logo	match2	qval2	match2_logo
de novo #647	100				Zfp661	2.248880e-03		Ctcfl	2.348340e-02		Ctcf	4.838120e-02	
de novo #648	58				Zfp661	1.693020e-03		Ctcfl	7.864050e-03		Ctcf	2.163480e-02	
de novo #649	304				Sp2	2.730540e-03		Klf6	9.844340e-03		Sp3	1.028190e-02	
de novo #650	39				Nfya	1.711190e-01		Nfyc	1.711190e-01		Nfyb	2.019400e-01	
de novo #651	55				Nfia	7.061070e-01		Insm1	7.061070e-01		Nfix	9.903440e-01	
de novo #652	107				Rarg	2.864390e-02		Hnf4g	2.864390e-02		Nr2c2	2.864390e-02	
de novo #653	44				Gata3	9.471880e-01		Hmg20a	9.471880e-01		Hmg20b	0.947188	
de novo #654	100				Crebl2	1.630660e-01		Atf7	1.630660e-01		Creb5	1.630660e-01	
de novo #655	139				Crebl2	2.215410e-01		Atf7	2.215410e-01		Creb5	2.215410e-01	
de novo #656	61				Gmeb2	8.074260e-02		Gmeb1	8.074260e-02		Jdp2	2.559810e-01	
de novo #657	2687				Creb3	3.008610e-04		Creb5	3.008610e-04		Gm28047	3.834930e-04	
de novo #658	2465				Creb3	1.738010e-04		Creb5	1.738010e-04		Atf7	1.738010e-04	
de novo #659	3329				Jdp2	1.183660e-04		Crebl2	3.466950e-04		Atf7	0.000347	
de novo #660	126				Atf7	1.409430e-01		Crebl2	1.409430e-01		Creb5	1.759050e-01	
de novo #661	3300				Atf7	1.740280e-04		Creb5	1.740280e-04		Creb3	1.740280e-04	

pattern	num_seqlets	modisco_cwm_fwd	modisco_cwm_rev	delta_effects	match0	qval0	match0_logo	match1	qval1	match1_logo	match2	qval2	match2_logo
de novo #662	3645				Atf7	1.986230e-04		Creb5	1.986230e-04		Creb3	1.986230e-04	
de novo #663	3645				Atf7	1.495860e-04		Creb3	1.495860e-04		Creb5	1.495860e-04	
de novo #664	3224				Jdp2	1.253070e-04		Crem	3.461900e-04		Atf7	0.000369	
de novo #665	48				Atf7	4.217430e-03		Jdp2	4.217430e-03		Crem	4.217430e-03	
de novo #666	26				Atf7	6.279400e-04		Jdp2	6.279400e-04		Gm28047	2.274260e-03	
de novo #667	114				Atf7	1.354580e-01		Crebl2	1.354580e-01		Creb5	0.152789	
de novo #668	4347				Atf7	1.852520e-04		Creb3	1.852520e-04		Creb5	1.852520e-04	
de novo #669	2414				Creb3	1.544650e-04		Creb5	1.544650e-04		Gm28047	1.989920e-04	
de novo #670	2481				Jdp2	2.351460e-04		Creb5	2.351460e-04		Atf7	2.351460e-04	
de novo #671	3103				Gm28047	1.276000e-04		Atf7	1.276000e-04		Creb3	1.276000e-04	
de novo #672	2430				Creb5	2.752930e-04		Atf7	2.752930e-04		Creb3	2.752930e-04	
de novo #673	128				Crebl2	1.560330e-01		Atf7	1.560330e-01		Creb5	1.699980e-01	
de novo #674	53				Gm28047	8.561760e-04		Creb1	8.561760e-04		Creb3	8.561760e-04	
de novo #675	44				Gm28047	6.356840e-04		Creb3	1.161210e-03		Atf2	6.908190e-03	
de novo #676	1220				Gata6	2.187460e-02		Gata1	2.187460e-02		Tal1	0.036457	

pattern	num_seqlets	modisco_cwm_fwd	modisco_cwm_rev	delta_effects	match0	qval0	match0_logo	match1	qval1	match1_logo	match2	qval2	match2_logo
de novo #677	21				Gata1	2.720950e-01		Gata2	2.720950e-01		Gata3	0.441625	
de novo #678	135				Gata6	4.968210e-02		Gata1	4.968210e-02		Tal1	7.756850e-02	
de novo #679	1443				Gata6	2.153830e-02		Gata1	2.153830e-02		Tal1	3.589650e-02	
de novo #680	33				Nr4a2	2.985280e-03		Nr4a3	2.985280e-03		Rarg	0.224949	
de novo #681	29				Nr4a2	2.389060e-01		Nr4a3	2.389060e-01		Nr1d1	8.028310e-01	
de novo #682	141				Hoxc10	4.271370e-02		Hoxd11	4.271370e-02		Hoxd10	0.042714	
de novo #683	208				Sall4	7.024600e-02		Gli3	5.846140e-01		Gli1	0.584614	
de novo #684	140				Sall4	1.127000e-01		Gli3	5.747630e-01		Gli1	5.747630e-01	
de novo #685	91				Zfp287	3.388250e-01		Irf1	3.388250e-01		Stat1	3.388250e-01	
de novo #686	403				Zfp287	8.450120e-10		Prdm6	1.102250e-07		Zfp422	9.605520e-05	
de novo #687	378				Zfp287	3.920800e-04		Foxj1	4.457560e-02		Stat1	7.197330e-02	
de novo #688	138				Zfp287	3.839660e-01		Irf4	3.839660e-01		Irf5	0.383966	
de novo #689	115				Zfp287	3.861030e-02		Stat1	6.001900e-02		Prdm6	6.001900e-02	
de novo #690	94				Zfp287	3.139150e-01		Stat1	3.139150e-01		Irf1	3.139150e-01	
de novo #691	88				Zfp287	2.753550e-02		Zfp879	3.066570e-01		Hmg20a	4.312980e-01	

pattern	num_seqlets	modisco_cwm_fwd	modisco_cwm_rev	delta_effects	match0	qval0	match0_logo	match1	qval1	match1_logo	match2	qval2	match2_logo
de novo #692	646				Zfp287	1.125750e-07		Zfp422	2.257130e-06		Prdm6	2.638270e-06	
de novo #693	547				Zfp422	2.188180e-05		Zfp287	1.587070e-04		Prdm6	1.289440e-03	
de novo #694	101				Stat1	3.652300e-02		Zfp287	9.309640e-02		Irf1	9.309640e-02	
de novo #695	3652				Zfp248	9.154430e-01		Zbtb32	9.154430e-01		Rhox11	1.000000e+00	
de novo #696	4560				Rhox11	7.095720e-01		Zbtb32	7.095720e-01		Zfp248	7.095720e-01	
de novo #697	212				Zfp146	1.000000e+00		Zbtb22	1.000000e+00		Zfp296	1.000000e+00	
de novo #698	4805				Zbtb32	4.248070e-01		Zfp248	7.213790e-01		Zfp146	9.851350e-01	
de novo #699	1567				Foxp2	1.000000e+00		Zfp146	1.000000e+00		Zbtb22	1.000000e+00	
de novo #700	1313				Zbtb22	1.000000e+00		Zfp146	1.000000e+00		NaN	NaN	
de novo #701	1469				Zbtb22	1.000000e+00		Zfp879	1.000000e+00		Zscan4b	1.000000e+00	
de novo #702	2447				Zfp248	3.221570e-01		Rhox11	1.000000e+00		NaN	NaN	
de novo #703	3090				Zfp146	1.000000e+00		Zfp296	1.000000e+00		Zfp322a	1.000000e+00	
de novo #704	729				Mef2c	6.549350e-01		Zfp422	6.549350e-01		Onecut3	6.549350e-01	
de novo #705	481				Irf1	1.187770e-04		Stat2	3.373750e-03		Stat1	3.373750e-03	
de novo #706	2512				Irf1	1.075050e-10		Stat2	2.192860e-05		Stat1	8.868950e-05	

pattern	num_seqlets	modisco_cwm_fwd	modisco_cwm_rev	delta_effects	match0	qval0	match0_logo	match1	qval1	match1_logo	match2	qval2	match2_logo
de novo #707	1756				Irf1	1.631840e-09		Stat2	8.885310e-05		Prdm1	1.465280e-04	
de novo #708	1401				Irf1	6.291360e-11		Prdm1	6.664970e-05		Irf8	2.958010e-04	
de novo #709	2251				Irf1	3.349870e-11		Stat2	2.578790e-05		Stat1	5.687980e-05	
de novo #710	424				Irf1	4.706910e-08		Prdm1	7.865050e-06		Stat2	2.997220e-05	
de novo #711	1766				Irf1	7.599320e-11		Stat2	2.415010e-05		Stat1	8.169800e-05	
de novo #712	412				Irf1	7.060990e-07		Prdm1	9.388650e-06		Stat2	2.203840e-04	
de novo #713	3072				Irf1	9.290650e-11		Stat2	1.804560e-05		Stat1	0.000033	
de novo #714	1928				Irf1	1.947380e-11		Prdm1	1.112800e-04		Irf8	2.801400e-04	
de novo #715	67				Irf1	1.782060e-01		Irf4	1.782060e-01		Stat1	1.832100e-01	
de novo #716	1580				Irf1	7.411170e-10		Prdm1	6.406950e-05		Irf8	2.547900e-04	
de novo #717	502				Irf1	3.298400e-07		Prdm1	5.266450e-06		Stat2	0.000134	
de novo #718	1335				Atf2	5.579790e-03		Gm28047	5.041290e-02		Creb5	0.050413	
de novo #719	871				Atf2	9.764710e-04		Gm28047	2.563250e-02		Atf7	2.563250e-02	
de novo #720	453				Atf2	6.145910e-03		Gm28047	5.211620e-02		Atf7	0.052116	
de novo #721	1487				Zfp143	1.699630e-17		Thap11	3.119170e-17		Six5	0.000027	

pattern	num_seqlets	modisco_cwm_fwd	modisco_cwm_rev	delta_effects	match0	qval0	match0_logo	match1	qval1	match1_logo	match2	qval2	match2_logo
de novo #722	1472				Zfp143	1.595050e-19		Thap11	3.325840e-19		Six5	3.872540e-05	
de novo #723	22				Thap11	2.717160e-08		Zfp143	1.265700e-07		Six5	2.017030e-03	
de novo #724	1414				Zfp143	1.164780e-17		Thap11	7.870210e-17		Six5	3.853210e-05	
de novo #725	1439				Zfp143	1.246610e-16		Thap11	1.833010e-16		Six5	2.435710e-05	
de novo #726	47				Thap11	1.958490e-10		Zfp143	3.923920e-10		Six5	2.736100e-03	
de novo #727	1425				Zfp143	2.561840e-17		Thap11	1.376230e-16		Six5	4.421530e-05	
de novo #728	1407				Zfp143	1.869390e-17		Thap11	1.070110e-16		Six5	3.799470e-05	
de novo #729	1402				Zfp143	2.021880e-18		Thap11	1.902910e-17		Six5	1.311270e-05	
de novo #730	1460				Zfp143	3.671010e-17		Thap11	6.185520e-17		Six5	4.120720e-05	
de novo #731	1429				Thap11	1.011100e-17		Zfp143	1.994630e-17		Six5	5.003290e-05	
de novo #732	1475				Thap11	8.565070e-20		Zfp143	1.831300e-19		Six5	3.711980e-05	
de novo #733	47				Zfp143	2.714530e-03		Stat3	2.714530e-03		Stat4	2.714530e-03	
de novo #734	1475				Zfp143	2.394360e-17		Thap11	2.394360e-17		Six5	3.536280e-05	
de novo #735	1427				Zfp143	4.522780e-17		Thap11	4.522780e-17		Six5	0.000018	
de novo #736	35				Nfix	1.000000e+00		Nfia	1.000000e+00		Rfx4	1.000000e+00	

pattern	num_seqlets	modisco_cwm_fwd	modisco_cwm_rev	delta_effects	match0	qval0	match0_logo	match1	qval1	match1_logo	match2	qval2	match2_logo
de novo #737	101				Sp2	8.580790e-03		Mxd1	1.358930e-02		Mxi1	1.358930e-02	
de novo #738	50				Nrf1	9.726960e-03		Zfp566	1.313810e-01		Elf1	0.131381	
de novo #739	70				Sp2	2.083390e-06		Sp3	2.162430e-05		Wt1	1.138100e-03	
de novo #740	622				Zbtb14	3.254080e-01		Nrf1	3.254080e-01		Zfp449	8.052930e-01	
de novo #741	35				Sp2	1.499730e-03		Sp3	2.680200e-03		Zfx	8.533670e-03	
de novo #742	64				Sp2	1.122600e-03		Zfp64	3.250040e-03		Zfx	3.250040e-03	
de novo #743	19564				Sp2	1.078940e-02		Sp3	1.902520e-02		Zfx	1.919390e-02	
de novo #744	73				Nrf1	1.686340e-02		Sp2	1.007180e-01		Sp3	1.007180e-01	
de novo #745	940				Zbtb14	3.075790e-01		Nrf1	3.075790e-01		Egr2	6.810890e-01	
de novo #746	68				Zfp692	1.024550e-01		Hic2	1.119330e-01		Sp2	1.119330e-01	
de novo #747	4844				Zfx	5.233010e-04		Sp2	4.231530e-03		Mxd1	4.835150e-03	
de novo #748	26				Sp2	7.886600e-05		Sp3	1.213960e-03		Mxd1	1.032660e-02	
de novo #749	930				Nrf1	2.379610e-01		Zbtb14	2.379610e-01		Egr2	7.494450e-01	
de novo #750	824				Nrf1	2.521350e-01		Zbtb14	2.521350e-01		Egr2	7.748480e-01	
de novo #751	662				Nrf1	1.954190e-01		Zbtb14	1.954190e-01		Egr2	0.785539	

pattern	num_seqlets	modisco_cwm_fwd	modisco_cwm_rev	delta_effects	match0	qval0	match0_logo	match1	qval1	match1_logo	match2	qval2	match2_logo
de novo #752	1932				Mlxipl	4.420390e-04		Tfeb	1.318980e-03		Usf1	1.545940e-03	
de novo #753	22				Tfe3	4.317240e-04		Tfeb	7.492260e-04		Tfec	7.492260e-04	
de novo #754	1746				Mlxipl	4.850630e-04		Tfeb	1.428790e-03		Tfe3	1.624080e-03	
de novo #755	2245				Usf1	3.547330e-04		Tfeb	7.772760e-04		Tfe3	1.098500e-03	
de novo #756	2060				Tfeb	2.633380e-05		Usf1	3.950070e-05		Arntl	1.586110e-04	
de novo #757	2589				Usf1	1.337120e-05		Tfeb	2.627250e-05		Arntl	3.011600e-04	
de novo #758	1708				Usf1	1.272600e-04		Tfeb	1.272600e-04		Arntl	0.000194	
de novo #759	1880				Tfeb	5.485810e-05		Usf1	8.228710e-05		Arntl	2.200540e-04	
de novo #760	48				Tfeb	1.783560e-03		Mxd1	1.783560e-03		Mxi1	1.783560e-03	
de novo #761	1969				Tfeb	1.100490e-03		Usf1	1.283900e-03		Mlxipl	1.447250e-03	
de novo #762	58				Tfeb	7.865160e-04		Mitf	4.018400e-03		Tfe3	4.018400e-03	
de novo #763	2626				Tfeb	1.241240e-03		Usf1	1.448110e-03		Mlxipl	1.499320e-03	
de novo #764	2229				Usf1	2.529390e-05		Tfeb	4.544370e-05		Arntl	1.402440e-04	
de novo #765	2764				Usf1	7.164380e-04		Tfe3	7.164380e-04		Tfeb	7.164380e-04	
de novo #766	2879				Usf1	6.661910e-05		Tfeb	9.534550e-05		Arntl	0.000288	

pattern	num_seqlets	modisco_cwm_fwd	modisco_cwm_rev	delta_effects	match0	qval0	match0_logo	match1	qval1	match1_logo	match2	qval2	match2_logo
de novo #767	49				Maf	9.446240e-01		Hnf4g	9.446240e-01		Hnf4a	9.446240e-01	
de novo #768	37				Tfap4	8.656480e-01		Figla	8.656480e-01		Maf	8.656480e-01	
de novo #769	51				Tfap4	8.494110e-01		Maf	8.494110e-01		Hnf4g	8.494110e-01	
de novo #770	30				Tfap4	9.407930e-01		Maf	1.000000e+00		Figla	1.000000	
de novo #771	29				Tfap4	8.134290e-01		Maf	1.000000e+00		Hnf4a	1.000000e+00	
de novo #772	39				Tfap4	8.337870e-01		Figla	1.000000e+00		Maf	1.000000	
de novo #773	144				Zfp513	1.000000e+00		Lmx1a	1.000000e+00		Dmrtc2	1.000000	
de novo #774	217				Hoxc10	2.034690e-01		Hoxa6	2.034690e-01		Hoxb8	2.034690e-01	
de novo #775	1256				Zfp692	1.402370e-01		Glis3	1.402370e-01		Glis1	1.402370e-01	
de novo #776	29				ENSMUSG00000097126	3.948810e-01		Rhox8	3.948810e-01		Hsf1	4.193210e-01	
de novo #777	2011				Hoxd11	6.137180e-01		ENSMUSG00000097126	6.137180e-01		Rhox8	6.137180e-01	
de novo #778	25				ENSMUSG00000097126	6.929790e-01		Rhox8	6.929790e-01		Six5	1.000000e+00	
de novo #779	95				Rfx1	2.730600e-08		Rfx3	4.100160e-07		Rfx5	4.100160e-07	
de novo #780	593				Rfx1	3.755350e-08		Rfx2	4.273450e-07		Rfx3	4.273450e-07	
de novo #781	496				Rfx1	6.805510e-09		Rfx3	2.102160e-08		Rfx5	2.102160e-08	

pattern	num_seqlets	modisco_cwm_fwd	modisco_cwm_rev	delta_effects	match0	qval0	match0_logo	match1	qval1	match1_logo	match2	qval2	match2_logo
de novo #782	517				Rfx1	6.270490e-09		Rfx3	5.771450e-08		Rfx5	5.771450e-08	
de novo #783	87				Rfx1	2.712800e-08		Rfx3	5.312380e-08		Rfx5	5.539100e-08	
de novo #784	74				Rfx1	1.599270e-08		Rfx3	1.599270e-08		Rfx5	3.704350e-08	
de novo #785	530				Rfx1	1.547070e-08		Rfx3	6.614360e-08		Rfx5	6.614360e-08	
de novo #786	112				Gli1	1.000000e+00		Zfp558	1.000000e+00		Zfp212	1.000000e+00	
de novo #787	400				Lcorl	1.125590e-01		Lcor	1.125590e-01		Mef2a	4.576510e-01	
de novo #788	319				Zfx	3.500380e-02		Klf3	6.164530e-01		Sp2	6.164530e-01	
de novo #789	208				Zfx	4.681980e-01		Klf3	4.681980e-01		Tead4	4.681980e-01	
de novo #790	487				Klf4	5.646680e-01		Klf7	5.646680e-01		Klf12	5.646680e-01	
de novo #791	316				Klf3	2.266030e-01		Klf5	2.266030e-01		Zfx	3.282680e-01	
de novo #792	1299				Zfx	2.051630e-04		Sp2	5.288080e-03		Klf3	5.288080e-03	
de novo #793	278				Zfx	1.000000e+00		Trp63	1.000000e+00		Klf5	1.000000e+00	
de novo #794	1231				Ddit3	1.000000e+00		Jdp2	1.000000e+00		Atf4	1.000000e+00	
de novo #795	93				Msantd3	1.000000e+00		Zfp110	1.000000e+00		Zfp691	1.000000e+00	
de novo #796	37				Hoxc12	1.000000e+00		Hoxd12	1.000000e+00		Hoxc11	1.000000	

pattern	num_seqlets	modisco_cwm_fwd	modisco_cwm_rev	delta_effects	match0	qval0	match0_logo	match1	qval1	match1_logo	match2	qval2	match2_logo
de novo #797	100				Zbtb43	1.000000e+00		Aire	1.000000e+00		Ovol1	1.000000e+00	
de novo #798	13335				Zfp213	5.572320e-01		Smad1	5.572320e-01		Smad5	5.572320e-01	
de novo #799	33				Foxi2	5.109770e-03		Foxi3	5.109770e-03		Foxg1	8.589870e-03	
de novo #800	28				Foxi2	2.626300e-03		Foxi3	2.626300e-03		Foxg1	0.009708	
de novo #801	40				Foxi2	4.287320e-03		Foxi3	4.287320e-03		Foxg1	0.014429	
de novo #802	35				Foxi2	3.550630e-02		Foxi3	3.550630e-02		Foxg1	5.049670e-02	
de novo #803	37				Foxi2	2.673450e-03		Foxi3	2.673450e-03		Foxg1	4.339960e-03	
de novo #804	1409				Mbtps2	2.020200e-06		Yy1	2.020200e-06		Yy2	2.020200e-06	
de novo #805	983				Mbtps2	3.128790e-06		Yy1	3.128790e-06		Yy2	3.128790e-06	
de novo #806	1307				Mbtps2	2.219290e-06		Yy1	2.219290e-06		Yy2	2.219290e-06	
de novo #807	340				Taf1	2.600770e-04		Mbtps2	1.378030e-03		Yy1	1.378030e-03	
de novo #808	1207				Mbtps2	2.934530e-06		Yy1	2.934530e-06		Yy2	2.934530e-06	
de novo #809	1371				Mbtps2	2.753180e-06		Yy1	2.753180e-06		Yy2	2.753180e-06	
de novo #810	1359				Mbtps2	2.061760e-06		Yy1	2.061760e-06		Yy2	0.000002	
de novo #811	1560				Mbtps2	1.278130e-06		Yy1	1.278130e-06		Yy2	1.278130e-06	

pattern	num_seqlets	modisco_cwm_fwd	modisco_cwm_rev	delta_effects	match0	qval0	match0_logo	match1	qval1	match1_logo	match2	qval2	match2_logo
de novo #812	1447				Mbtps2	1.598220e-06		Yy1	1.598220e-06		Yy2	1.598220e-06	
de novo #813	348				Taf1	3.463260e-05		Mbtps2	3.463260e-05		Yy1	3.463260e-05	
de novo #814	444				Taf1	1.481250e-04		Mbtps2	1.481250e-04		Yy1	1.481250e-04	
de novo #815	1322				Mbtps2	2.790440e-06		Yy1	2.790440e-06		Yy2	2.790440e-06	
de novo #816	1812				Mbtps2	1.044780e-06		Yy1	1.044780e-06		Yy2	1.044780e-06	
de novo #817	1203				Mbtps2	4.837490e-06		Yy1	4.837490e-06		Yy2	4.837490e-06	
de novo #818	1129				Mbtps2	3.719150e-06		Yy1	3.719150e-06		Yy2	0.000004	
de novo #819	112				Mbtps2	3.609060e-05		Yy1	3.609060e-05		Yy2	3.609060e-05	
de novo #820	1359				Zfp692	1.271090e-01		Glis3	1.271090e-01		Glis1	1.271090e-01	
de novo #821	926				Dnmt1	3.771670e-01		Sp1	3.771670e-01		Klf11	3.771670e-01	
de novo #822	829				Zbtb32	3.367220e-01		Lin28a	3.367220e-01		Lin28b	3.367220e-01	
de novo #823	103				Klf15	5.209350e-02		Patz1	2.607040e-01		Wt1	0.308157	
de novo #824	745				Ddit3	1.000000e+00		Jdp2	1.000000e+00		Atf4	1.000000e+00	
de novo #825	623				Cebpb	7.806750e-05		Cebpg	1.851200e-04		Dbp	6.527810e-04	
de novo #826	839				Cebpg	5.629310e-05		Cebpb	1.151730e-04		Cebpa	3.713930e-04	

pattern	num_seqlets	modisco_cwm_fwd	modisco_cwm_rev	delta_effects	match0	qval0	match0_logo	match1	qval1	match1_logo	match2	qval2	match2_logo
de novo #827	963				Cebpg	2.508040e-06		Cebpb	5.029810e-06		Cebpa	1.928110e-04	
de novo #828	1615				Cebpb	4.272830e-06		Cebpg	3.076200e-05		Cebpa	0.000372	
de novo #829	344				Cebpb	1.596370e-04		Dbp	1.596370e-04		Hlf	3.172690e-04	
de novo #830	840				Cebpb	8.140910e-07		Cebpg	9.533170e-06		Cebpa	9.032300e-05	
de novo #831	188				Tef	2.803680e-03		Dbp	2.803680e-03		Hlf	2.803680e-03	
de novo #832	220				Zbtb7c	2.415940e-02		Ctcfl	9.940760e-02		Zfp661	9.940760e-02	
de novo #833	827				Ddit3	1.000000e+00		Jdp2	1.000000e+00		Fosl1	1.000000e+00	
de novo #834	92				Rfx7	6.920220e-02		Rfx6	6.920220e-02		Rfx4	7.845180e-02	
de novo #835	189				Rfx7	8.145620e-02		Rfx4	8.145620e-02		Rfx8	8.145620e-02	
de novo #836	72				Rfx6	1.250620e-01		Rfx4	1.250620e-01		Rfx7	1.250620e-01	
de novo #837	52				Rfx6	2.616870e-03		Ovol1	3.241280e-01		Zfp740	5.138190e-01	
de novo #838	318				Rfx7	8.290820e-02		Rfx6	8.290820e-02		Rfx4	1.302900e-01	
de novo #839	391				Zbtb33	6.472020e-05		Srebf2	4.317450e-01		E2f8	1.000000e+00	
de novo #840	397				Zbtb33	3.640190e-05		Srebf2	3.826190e-01		E2f8	1.000000	
de novo #841	387				Zbtb33	2.480140e-05		Srebf2	3.355080e-01		E2f8	1.000000e+00	

pattern	num_seqlets	modisco_cwm_fwd	modisco_cwm_rev	delta_effects	match0	qval0	match0_logo	match1	qval1	match1_logo	match2	qval2	match2_logo
de novo #842	375				Zbtb33	1.125320e-04		Srebf2	4.009510e-01		E2f8	1.000000e+00	
de novo #843	373				Zbtb33	5.158180e-05		Srebf2	3.630690e-01		E2f8	1.000000e+00	
de novo #844	353				Zbtb33	3.706960e-05		Srebf2	3.678380e-01		E2f8	1.000000e+00	
de novo #845	350				Zbtb33	3.892130e-05		Srebf2	3.999330e-01		E2f8	1.000000e+00	
de novo #846	364				Zbtb33	1.763280e-04		Srebf2	4.136620e-01		E2f8	1.000000e+00	
de novo #847	415				Zbtb33	2.661440e-05		Srebf2	4.093920e-01		E2f8	1.000000e+00	
de novo #848	397				Zbtb33	1.012100e-04		Srebf2	4.161420e-01		E2f8	1.000000	
de novo #849	392				Zbtb33	7.258180e-05		Srebf2	3.786330e-01		E2f8	1.000000e+00	
de novo #850	405				Zbtb33	1.666840e-04		Srebf2	4.274000e-01		E2f8	1.000000e+00	
de novo #851	48				Elk1	1.477420e-01		Elf1	3.441610e-01		Ascl1	3.441610e-01	
de novo #852	1112				Ddit3	1.000000e+00		Jdp2	1.000000e+00		Atf4	1.000000	
de novo #853	498				Elk1	3.041220e-01		Erg	3.041220e-01		Erf	3.041220e-01	
de novo #854	360				Elk1	2.776610e-01		Erg	2.776610e-01		Erf	0.277661	
de novo #855	27				Sox3	5.060310e-01		Sox6	5.060310e-01		Tcf7l1	5.060310e-01	
de novo #856	35				Sox3	5.494680e-01		Sox6	6.614350e-01		Cdx2	0.721729	

pattern	num_seqlets	modisco_cwm_fwd	modisco_cwm_rev	delta_effects	match0	qval0	match0_logo	match1	qval1	match1_logo	match2	qval2	match2_logo
		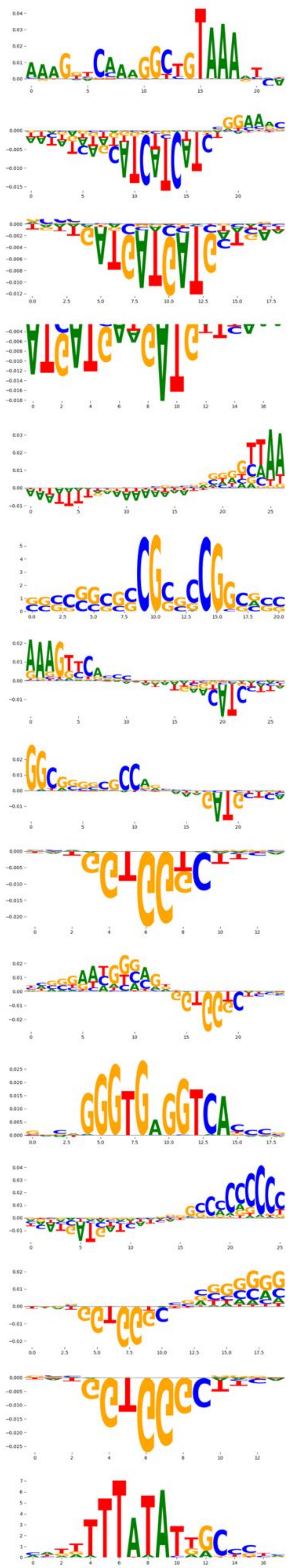	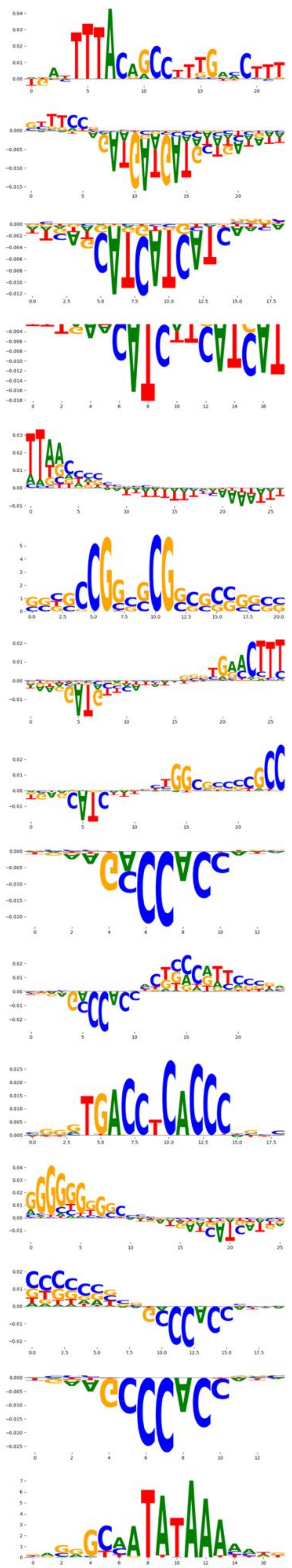	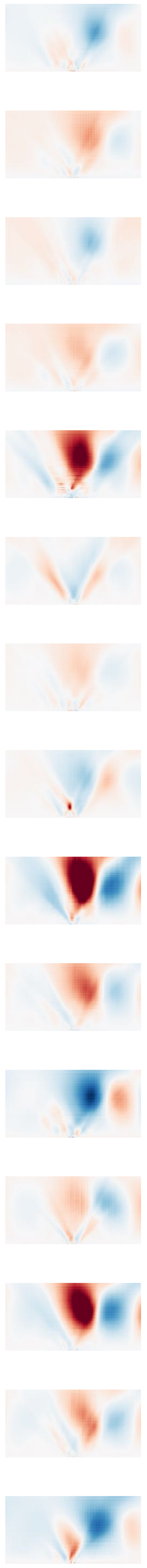			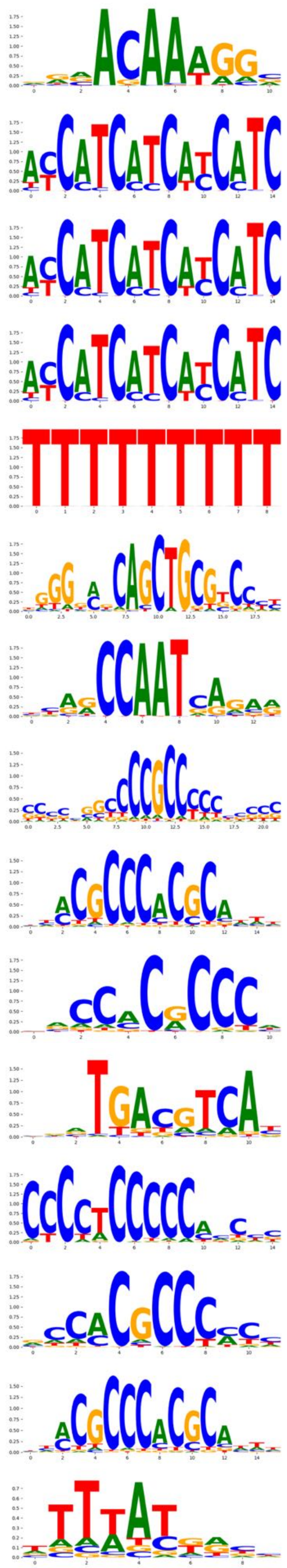			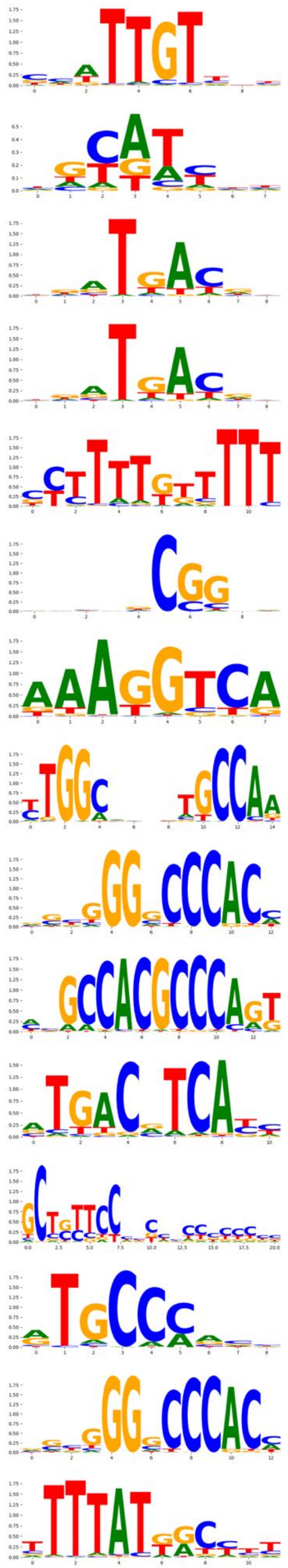			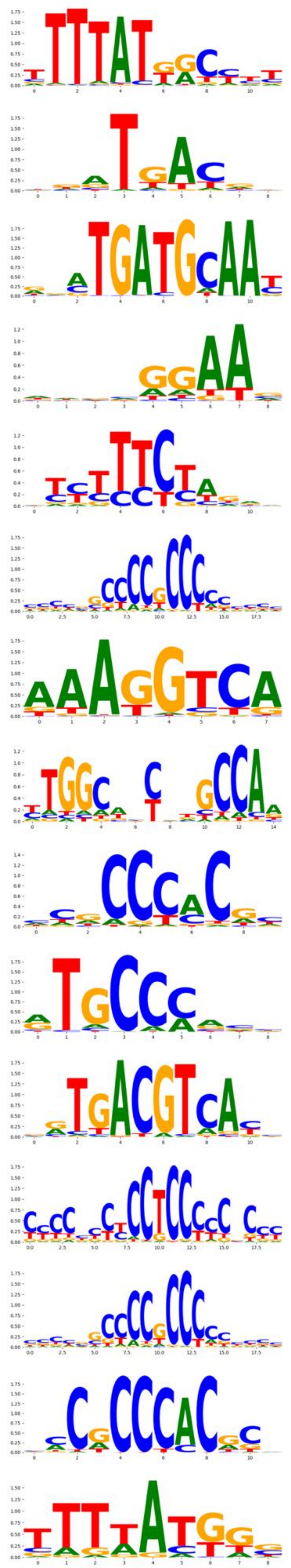
de novo #857	69				Zfp513	2.839400e-01		Foxm1	1.000000e+00		Atf3	1.000000	
de novo #858	142				Zfp513	1.348040e-02		Atf3	4.195860e-01		Atf4	4.195860e-01	
de novo #859	67				Zfp513	3.640060e-03		Atf3	1.000000e+00		Spic	1.000000e+00	
de novo #860	40				Zfp422	1.243490e-01		Zfp182	6.001590e-01		Bcl6b	6.001590e-01	
de novo #861	198				Nhlh1	3.853760e-01		Dnmt1	7.342690e-01		Sp3	7.342690e-01	
de novo #862	96				Nfyc	3.438180e-01		Nr4a2	3.438180e-01		Nr4a3	3.438180e-01	
de novo #863	22				Sp2	4.915650e-01		Nfix	4.915650e-01		Nfia	4.915650e-01	
de novo #864	438				Egr4	1.172050e-01		Zfp692	1.172050e-01		Egr3	0.117205	
de novo #865	23				Klf2	6.158450e-01		Sp6	6.158450e-01		Hic2	0.615845	
de novo #866	52				Gm28047	5.163340e-02		Atf2	5.163340e-02		Creb3	5.163340e-02	
de novo #867	100				Zfp281	9.087940e-04		Zfp341	1.770330e-03		Wt1	1.876350e-03	
de novo #868	52				Sp9	1.307970e-01		Hic2	1.307970e-01		Sp3	0.130797	
de novo #869	320				Egr4	8.566680e-02		Zfp692	8.566680e-02		Egr2	8.566680e-02	
de novo #870	61				Hoxb9	2.965560e-01		Cdx2	2.965560e-01		Hoxd9	2.965560e-01	
de novo #871	39				Zfp1	7.326430e-01		Gm5294	7.326430e-01		Tbp	0.732643	

pattern	num_seqlets	modisco_cwm_fwd	modisco_cwm_rev	delta_effects	match0	qval0	match0_logo	match1	qval1	match1_logo	match2	qval2	match2_logo
de novo #872	257				Hoxb9	3.495340e-01		Cdx2	3.495340e-01		Tbp	3.495340e-01	
de novo #873	388				Cdx2	2.183960e-02		Hoxb9	4.119070e-02		Hoxc11	7.499810e-02	
de novo #874	292				Cdx2	2.017840e-01		Hoxb9	2.017840e-01		Hoxd9	2.017840e-01	
de novo #875	189				Sp2	3.820920e-03		Sp3	3.622310e-02		Klf5	3.622310e-02	
de novo #876	29				Klf15	3.172150e-03		Maz	7.417370e-03		Zfp566	7.417370e-03	
de novo #877	240				Zfp64	2.970940e-04		Sp2	2.970940e-04		Sp3	0.000627	
de novo #878	25				Sp2	1.334870e-02		Zfx	1.334870e-02		Sp3	0.014580	
de novo #879	195				Plagl1	1.381220e-01		Zfp692	5.487670e-01		Rxrb	5.487670e-01	
de novo #880	162				Zfp64	5.185630e-02		Zfp692	1.626580e-01		Zbtb7a	1.801650e-01	
de novo #881	35				Plagl1	2.327240e-01		Tfdp1	2.327240e-01		Tfdp2	2.327240e-01	
de novo #882	163				Zfp513	4.260840e-03		Tfap2c	1.000000e+00		Kmt2a	1.000000e+00	
de novo #883	238				Zfp513	2.433830e-03		Mbd2	1.000000e+00		Zbtb14	1.000000e+00	
de novo #884	92				Nfia	5.405600e-03		Nfix	4.785680e-02		Rfx2	0.070284	
de novo #885	126				Egr1	9.393120e-02		Egr3	9.393120e-02		Egr4	2.073930e-01	
de novo #886	84				Zfp641	2.379920e-02		Glis1	2.379920e-02		Glis3	2.379920e-02	

pattern	num_seqlets	modisco_cwm_fwd	modisco_cwm_rev	delta_effects	match0	qval0	match0_logo	match1	qval1	match1_logo	match2	qval2	match2_logo
de novo #887	69				Zfp641	5.822350e-02		Glis3	5.822350e-02		Glis1	0.058223	
de novo #888	42				Sp1	1.245120e-02		Klf16	1.245120e-02		Sp2	1.245120e-02	
de novo #889	31				Ctcf	9.469410e-02		Mbtps2	9.469410e-02		Yy1	9.469410e-02	
de novo #890	34				Ctcf	4.547620e-02		Mbtps2	4.547620e-02		Yy1	4.547620e-02	
de novo #891	60				Ctcf	6.624900e-02		Zfp383	6.003150e-01		Taf1	6.003150e-01	
de novo #892	69				Hic2	5.532840e-01		Klf16	1.000000e+00		Hic1	1.000000e+00	
de novo #893	346				Zbtb7b	1.089080e-02		Zbtb7a	2.129220e-02		Zfp641	2.129220e-02	
de novo #894	41				Foxm1	4.934880e-01		Atf4	1.000000e+00		Snai2	1.000000e+00	
de novo #895	36				Ctcfl	2.206550e-01		Patz1	2.206550e-01		Sp2	2.206550e-01	
de novo #896	145				Ctcfl	1.982930e-02		Tfap2c	1.712290e-01		Tfap2a	1.712290e-01	
de novo #897	205				Sp2	8.940130e-03		Ctcfl	8.940130e-03		Sp3	1.655340e-02	
de novo #898	23				Irf9	1.000000e+00		Zfp213	1.000000e+00		Zkscan17	1.000000e+00	
de novo #899	83				Ubp1	1.000000e+00		Hes6	1.000000e+00		Zbtb1	1.000000e+00	
de novo #900	52				Sp2	1.000000e+00		Irf9	1.000000e+00		Hes6	1.000000e+00	
de novo #901	681				Sox18	5.434120e-01		Sox7	5.434120e-01		Sox14	5.434120e-01	

pattern	num_seqlets	modisco_cwm_fwd	modisco_cwm_rev	delta_effects	match0	qval0	match0_logo	match1	qval1	match1_logo	match2	qval2	match2_logo
de novo #902	24				Dmrt2	8.686820e-01		Hoxc4	8.686820e-01		Hoxa4	8.686820e-01	
de novo #903	37				Foxg1	1.057650e-02		Foxi2	1.057650e-02		Foxi3	1.057650e-02	
de novo #904	166				Zfp513	1.569830e-01		NaN	NaN		NaN	NaN	
de novo #905	113				Zfp513	1.000000e+00		Zfp341	1.000000e+00		Klf15	1.000000	
de novo #906	64				Elk1	5.766790e-02		Elk4	5.766790e-02		ENSMUSG00000098546	5.766790e-02	
de novo #907	135				Zkscan17	1.000000e+00		Zbtb6	1.000000e+00		Mzf1	1.000000e+00	
de novo #908	90				Lcorl	1.000000e+00		Zfp740	1.000000e+00		Rreb1	1.000000	
de novo #909	58				Foxm1	1.000000e+00		Sox1	1.000000e+00		Nhlh1	1.000000	
de novo #910	219				Sp5	4.804700e-02		Sp3	4.804700e-02		Klf11	0.048047	
de novo #911	203				Hmg20a	2.487680e-01		Hmg20b	2.487680e-01		Foxp1	0.248768	
de novo #912	159				Zfp64	7.660710e-03		Zbtb6	8.819150e-02		Zbtb7a	8.819150e-02	
de novo #913	156				Zfp513	8.534520e-02		Ptf1a	3.872740e-01		Dnmt1	3.872740e-01	
de novo #914	90				Klf2	1.449740e-01		Klf8	1.449740e-01		Klf12	1.449740e-01	
de novo #915	27				Esrrb	2.360660e-01		Nr5a1	2.360660e-01		Esrra	2.360660e-01	
de novo #916	46				Nr5a1	4.169360e-01		Esrra	4.169360e-01		Zfp524	7.531790e-01	

pattern	num_seqlets	modisco_cwm_fwd	modisco_cwm_rev	delta_effects	match0	qval0	match0_logo	match1	qval1	match1_logo	match2	qval2	match2_logo
de novo #917	20				Nr5a1	3.176500e-01		Esrra	3.176500e-01		Zfp524	1.000000e+00	
de novo #918	61				Zfp423	1.000000e+00		Plagl1	1.000000e+00		Insm1	1.000000e+00	
de novo #919	38				Irf9	1.000000e+00		Onecut1	1.000000e+00		Zfp524	1.000000e+00	
de novo #920	98				Irf9	1.000000e+00		NaN	NaN		NaN	NaN	
de novo #921	25				Irf5	3.350280e-01		Irf4	3.350280e-01		Irf9	0.335028	
de novo #922	63				Sp2	1.000000e+00		Irf9	1.000000e+00		NaN	NaN	
de novo #923	24				Sp2	1.000000e+00		Irf9	1.000000e+00		Prdm14	1.000000	
de novo #924	44				Irf9	1.000000e+00		NaN	NaN		NaN	NaN	
de novo #925	50				Rorc	1.643900e-01		Gmeb2	1.643900e-01		Nr1d2	1.643900e-01	
de novo #926	53				Rorc	1.312700e-01		Nr1d2	1.312700e-01		Gmeb2	1.727260e-01	
de novo #927	55				Zfp513	5.154070e-01		Zbtb17	1.000000e+00		Rest	1.000000e+00	
de novo #928	34				Elk1	1.663850e-02		Elf1	1.663850e-02		Elf2	1.663850e-02	
de novo #929	99				Zfp740	9.086260e-01		Zic5	9.086260e-01		Pbx1	0.908626	
de novo #930	101				Hoxa7	1.000000e+00		Barhl2	1.000000e+00		Lcorl	1.000000e+00	
de novo #931	32				Zfp64	1.284770e-01		Usf1	3.030700e-01		Zfp770	3.842820e-01	

pattern	num_seqlets	modisco_cwm_fwd	modisco_cwm_rev	delta_effects	match0	qval0	match0_logo	match1	qval1	match1_logo	match2	qval2	match2_logo
de novo #932	51				Tfap2a	5.044140e-01		Tfap2d	5.044140e-01		Nr1i3	1.000000e+00	
de novo #933	28				Klf1	2.757340e-02		Trp63	2.757340e-02		Klf4	2.757340e-02	
de novo #934	212				Zfp182	1.000000e+00		Foxj1	1.000000e+00		Foxq1	1.000000e+00	
de novo #935	53				Zfp422	4.404100e-01		Hmga1	4.404100e-01		Hmga1rs1	0.440410	
de novo #936	92				Smad1	2.883630e-01		Smad5	2.883630e-01		Taf1	5.112820e-01	
de novo #937	85				Klf6	9.999260e-01		Klf5	9.999260e-01		Plag1	9.999260e-01	
de novo #938	213				Sp4	4.071480e-02		Klf11	5.380940e-02		E2f4	6.428610e-02	
de novo #939	24				Nhlh2	1.384520e-01		Runx3	7.255690e-01		Nhlh1	9.289600e-01	
de novo #940	36				Zbtb7b	1.378390e-01		Zfp423	1.378390e-01		Zbtb7a	1.378390e-01	
de novo #941	134				Atf4	3.530840e-01		Hoxa1	3.530840e-01		Msx3	3.530840e-01	
de novo #942	137				Sp6	5.934270e-02		Klf1	2.984300e-01		Klf2	0.298430	
de novo #943	48				Stat2	1.000000e+00		Stat1	1.000000e+00		Rorc	1.000000e+00	
de novo #944	39				Spic	1.853470e-01		Etv6	3.813000e-01		Etv3	0.494329	
de novo #945	37				Mxd1	2.085830e-04		Mxi1	2.085830e-04		Usf1	1.593050e-02	
de novo #946	50				Arntl	4.216840e-02		Tfe3	4.216840e-02		E2f6	4.216840e-02	

pattern	num_seqlets	modisco_cwm_fwd	modisco_cwm_rev	delta_effects	match0	qval0	match0_logo	match1	qval1	match1_logo	match2	qval2	match2_logo
		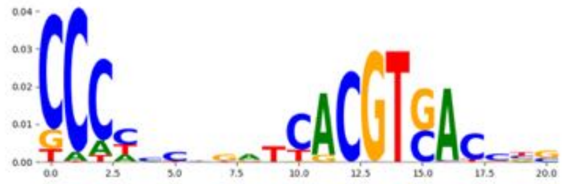	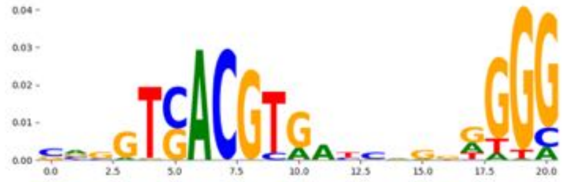	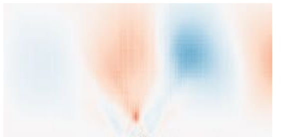			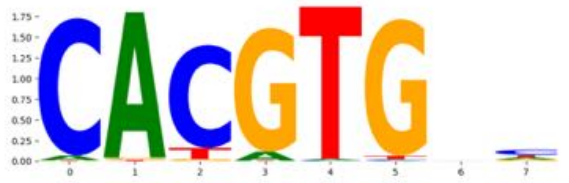			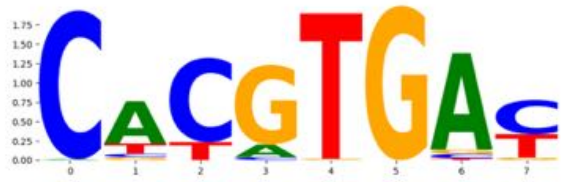			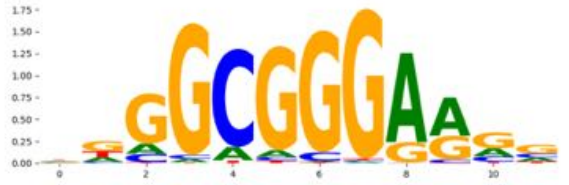
de novo #947	85	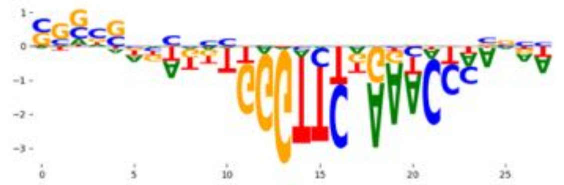	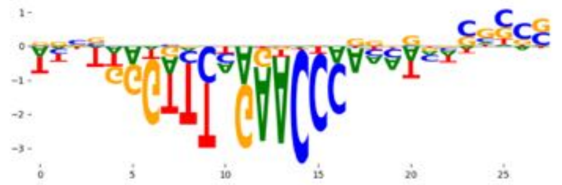	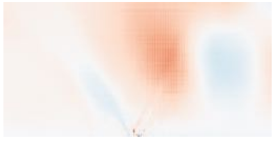	Zfp69	1.000000e+00	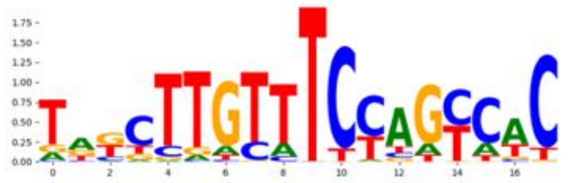	Zfp1	1.000000e+00	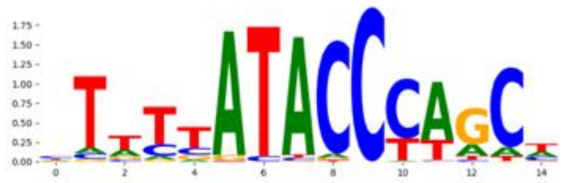	Tcf12	1.000000e+00	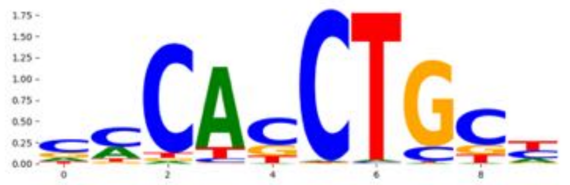
de novo #948	64	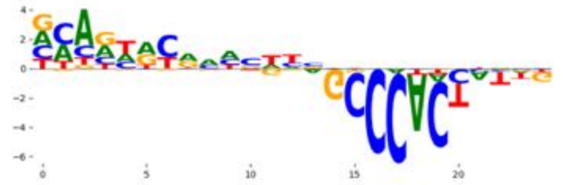	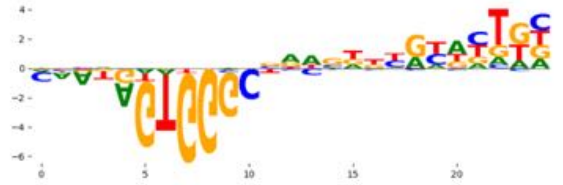	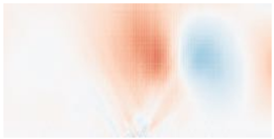	Hic2	2.435940e-03	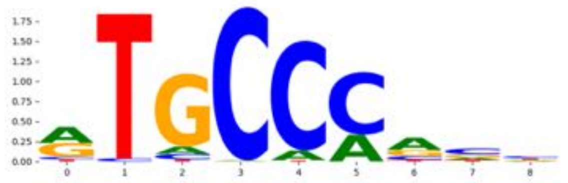	Hic1	1.441250e-02	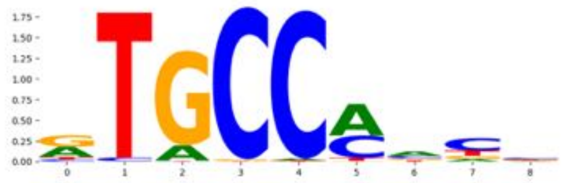	Zkscan6	8.077890e-01	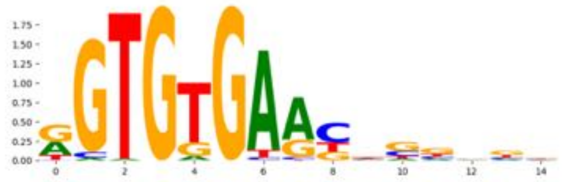
